# Kinetic and isothermal study of dye absorption using pre-treated natural fabrics using polyamine compounds

**DOI:** 10.1038/s41598-025-86012-z

**Published:** 2025-01-30

**Authors:** Eman M. Reda, Hanan A. Othman, Heba Ghazal, Ahmed G. Hassabo

**Affiliations:** 1 Textile Printing, Dyeing and Finishing Department, Faculty of Applied Arts, Tanta University, Tanta, Egypt; 2 Textile Printing, Dyeing and Finishing Department, Faculty of Applied Arts, Benha University, Benha, Egypt; 3 Finishing of Cellulose-based Fibres Department, National Research Centre, Pretreatment and Textile Research and Technology Institute, 33 El-Behouth St. (former El-Tahrir str.), Dokki, P.O. 12622, Giza, Egypt

**Keywords:** Polyethyleneimine, Chitosanm textile fabric, Kintec and isothermal, Dyeability, Chemistry, Materials science

## Abstract

The study examined the use of cationic polymers (Polyethyleneimine and chitosan) in treating fabrics like cotton, wool, and cotton/wool (70/30) to improve their dyeability and printability. The study examined factors such as dye concentration, time, and temperature for the dyeing process. Results showed that all dyed and printed fabrics treated with polyethyleneimine and chitosan increased color strength by significant percentages. Fastness properties, such as washing, rubbing, acidic and alkaline perspiration, and lightness, improved significantly. Fabric roughness, tensile strength, and elongation decreased by about 4-10% for each fabric. Additionally, the dyed and printed fabrics showed high resistance to bacteria and fungi. The study contributed to reducing chemicals used in traditional dyeing processes by a significant percentage, as the dyeing bath contained only the treated sample and dye solution. Furthermore kintic and isothermal study was investigated to explain the behaviour of treated fabrics for dye absorption.

## Introduction

In the dyeing process, about 10–15% of dyes are released into the aquatic environment, but when using reactive dyes, a higher percentage of such dyes cannot react anymore to cellulose fibers because of the side reaction of the hydrolysis during the dyeing process. Thus, roughly 20% of the original concentration of color dye is found in the reactive dye bath resulting in brightly colored effluent, and the existence of such dyes can be mutagenic and carcinogenic, causing significant harm to the human liver, digestive and central nervous system and affect the consistency of farming and underground water. Consequently, dyestuff effluents must be treated before they are released into the ecosystem^1^. On the other hand, because cotton, wool, and cotton/wool fibers are cellulosic and protein-based, the fabrics which are made from them are susceptible to being infected with bacteria, fungus, and mildew^2^.

One of the disadvantages found during dyeing of the cotton/wool blended fabric is the homogeneity of the formed color on the substrate as a result of the difference in the pH value appropriate for both cotton and wool material separately as cotton needs an alkaline medium, unlike wool which needs an acidic medium^3,4^.

Finishing is known that the fiber’s surface characteristics play a vital role in the aesthetic and functional properties of the fabrics that improve textile properties generally and helps to achieve desired properties, giving the textile its final commercial value and character^5^.

In recent years, polymers are now used widely instead of simple chemicals to improve multiple functional properties simultaneously, such as increasing absorbency, antimicrobial properties, and ultraviolet protection (UV) by any finishing or coating methods^6–8^.

Polyelectrolyte adsorption has been researched in-depth for the cellulosic and keratin fiber for approximately 40 years, Chitosan and polyethyleneimine are from these polyelectrolytes. Using polyethyleneimine (PEI) for fiber pre-treatment greatly favors the adsorption of coloring dyes in fiber and may be particularly interesting for the textile field as this increases the antimicrobial activity significantly^9^. As PEI has amino groups that exists in three forms (primary, secondary, and tertiary amines), each third or a quarter of nitrogen has its high densities at neutral pH^10^.

By looking for an eco-friendly process that can be carried out without toxic textile chemicals, chitosan is an excellent candidate for the eco-friendly textile industry, has a high Nitrogen Atom content, and is also a low cost abundant natural material that requires little processing^11,12^.

This study aimed to enhance natural fabric’s dyeability with natural dye (tea extract) and synthetic dyes (reactive and acid) using polycation, and to have higher color strength K/S and colorfastness properties furthermore to provide them with new functional properties such as antimicrobial protection.

## Experimental

### Materials

**Fig. 1:**
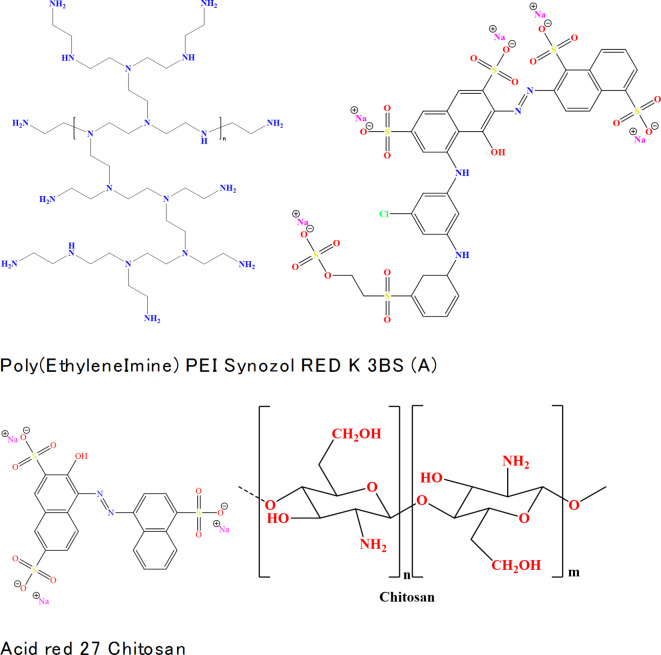
Chemical structure of PEI, Chitosan, Synozol RED K 3BS and Acid red 27.

In this study cotton (C, 100%; 225 g/m^2^) supplied by El-Nasr Spinning and Weaving Co., wool (W, 100%; 235 g/m^2^) company – mill-scoured and bleached cotton/wool 70/30 (CW, 70/30; 195 g/m^2^) were obtained from the Golden-Tex and used for the application. Polycation (PC) materials (Polyethyleneimine (PEI, water-free Mw = 25000 g/mol, Aldrich Co., see Fig. 1for chemical structure) – Chitosan (Ch) (low molecular weight, deacetylation 82.9% Dalton, Vanson Inc. Co. USA). Sodium carbonate (Na_2_CO_3_), methylene chloride (CH_2_Cl_2_), acetic acid (CH_3_COOH), sodium thiosulfate (Na_2_S_2_O_3_), hydrochloric acid (HCl), sodium chloride (NaCl), sodium hydroxide (NaOH), and non-ionic detergent were used in laboratory grade.

Reactive Dye (Syozol red k-3BS) and acid dye (Acid red 27) were kindly supplied from Newtrac company and their chemical structure is illustrated in Fig. 1. Dried tea leaves were purchased from the Egyptian local market.

### Methods

#### Preparation of tea leaves extract

Extraction of *tea* leaves was done according to previous work^13^. In brief, 20 g dried *tea* leaves were placed in 500 ml water in the Soxhlet system at boiling temperature for 24 h. The water extract was then concentrated using a rotary evaporator to 250 ml, and the extract was used without any purification.

#### Fabric pre-treatment

Before being treated with PEI or chitosan compounds, textile fabrics were washed to remove waxy components and impurities from the outer surface of the fibers. Cotton and cotton/wool fabrics were washed in a 2 g/L (Na_2_CO_3_) solution for 15 min, while wool fibers were washed in CH_2_Cl_2_ for 15 min, both at 40 °C, and then dried at room temperature.

#### Fabric treatment with polycation compounds

Chitosan and polyethyleneimine solutions with different concentrations (0.5, 1, 2, or 4%w/v) have been prepared by dissolving chitosan powder in 2% acetic acid solution under continuous stirring and dissolving PEI in distilled water under stirring. Washed fabrics were immersed in the solutions of each previous composite for about 20 min at 50 °C. The samples were then padded to a wet pick-up of 100% followed by drying for 5 min at 100 °C.

#### Dyeing procedure

Dyeing of the fabric samples was performed using synthetic and natural dyes (tea extract). Dyeing parameters were studied such as (i). Effect of polycation type and concentration (PEI, chitosan; (0.5, 1, 2, or 4% w/v)), (ii) temperature (30, 45, 60, 75, and 90 °C), and (iii) time (10, 20, 30 min.). dyeing process was done at1:30 LR, at pH 6.5 of the dye bath. dye concentration (0.5 g/L); dyeing temperature: 60 °C, Dyeing Time: 20 min, Finally, the dyed fabrics were rinsed with tap water and dried in an air oven at 100 °C for 5 min. and cured at 140 °C for 5 min^14–17^. curing step was done to ensuring the reaction between crosslinker and polymer and dye molecule as it need 140 C for complete the reaction as mentioned before^18^.

**Fig. 2:**
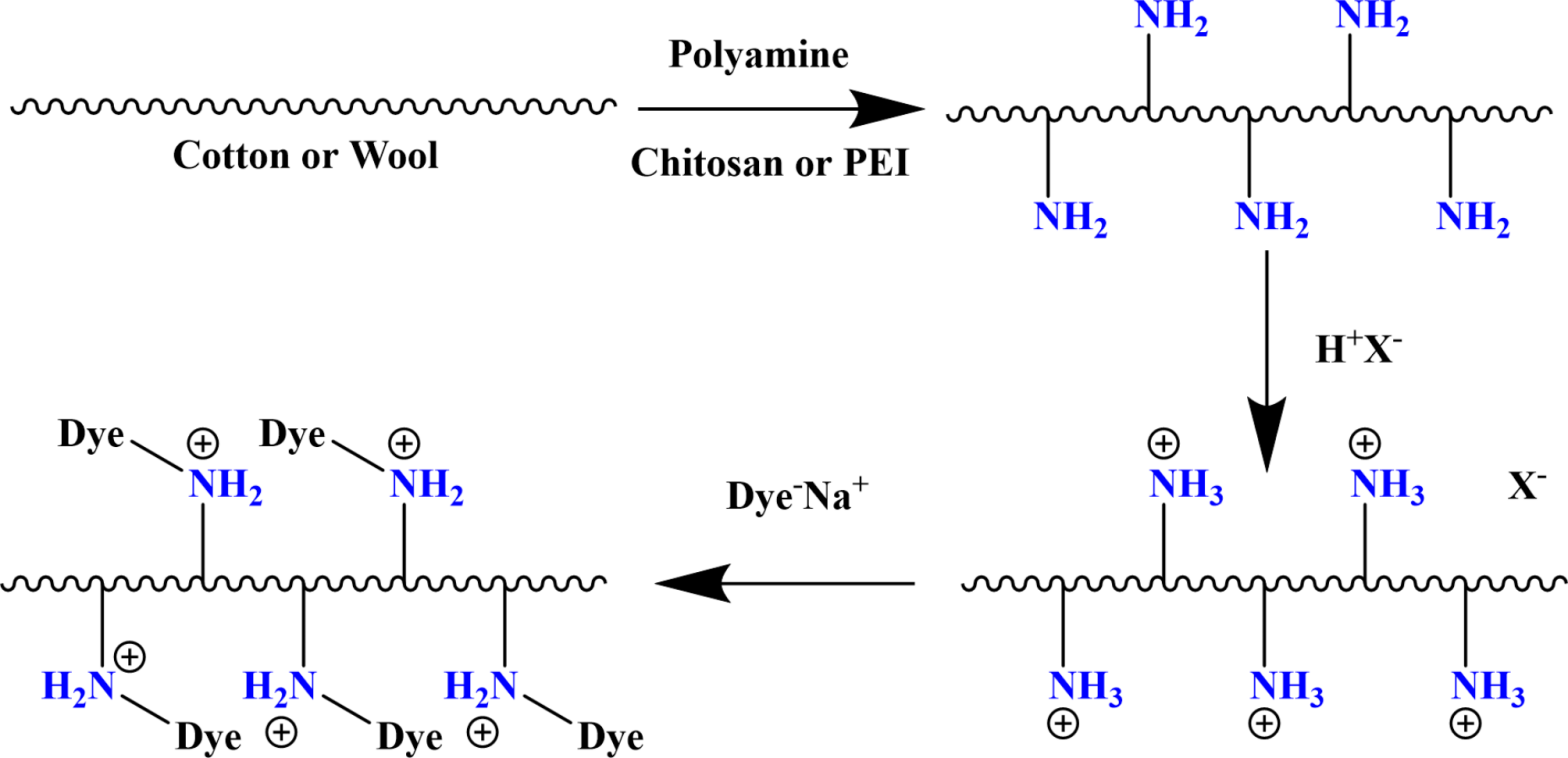
Mechanism of dyeing functionlysed surface with amine groups with Dyes.

However, the mechanisms of dyeing treated fabrics cotton, wool and their blend with both dyes are shown in Fig. 2. From Fig. 2, it can be seen that dye attachment onto both treated fabrics by ionic or salt links. Therefore, the hydrophilicity and dyeability of fabrics containing active amino groups on their surface were satisfactory, and provide multifunctional properties such as antistatic properties, environmental protection, ultraviolet protection antioxidant and antimicrobial activities, etc^19–21^. Owing these fabrics containing active sites of amino (-NH_2_), dye molecules interact chiefly with these fabrics by ionic bonding between electron-rich phenol ions present in dye molecules and the protonated amino groups of these fabrics (-NH_3_^+^) in the acidic medium to form hydrophobic dye–fiber interaction. In the alkaline medium, the active cationic sites of amino groups react covalently with hydroxyl groups from the dye bath and increased the dye uptake.

### Measurements

#### Determination of the amine content

The following method is used to determine the amine content of the prepared materials and/or fabrics: Weigh about 0.25 g of the sample and pour 40 ml of 0.1 M HCl (30 g NaCl + 10 g HCl in one liter, then standardized with Na_2_S_2_O_3_) into a 250 ml stoppered conical flask. The conical flasks were left overnight with occasional shaking and then, filtered and titrate the filtrate with phenolphthalein as an indicator against NaOH (0.05 M). The percentage of amino acids was estimated using the following equation:

amine contents % = $$\:\frac{{(V}_{B}-\:{V}_{s})\:\times\:\:{M}_{NaOH}}{{W}_{t}}\:\times\:100$$

which V_b_ is the Volume of NaOH consumed by the blank experiment, V_s_ is the Volume of NaOH consumed by the sample, M_NaOH_ = Molarity of NaOH, and W is the weight of the sample in H form.

#### Fourier Transform Infrared (FT-IR)

The FT-IR of the treated and untreated samples was evaluated using the JASCO spectrometer tester.

#### Color measurements

Coloration and spectral reflection values of the treated fibers were determined with a computer-based automated philtre spectrometer (Data Color Model 3890, Marl Co., Germany), and the color strength K/S of the dyed untreated and treated cotton fibers. The K/S values were calculated by using the Kubelka-Munk equation accordingly:^5,22–28^.

$$\frac{K}{S} = \frac{{\left( {1 - R} \right)^{2} }}{{2R}} - \frac{{\left( {1 - R} \right)^{2} }}{{2R_{0} }}$$

Where: K is representing the absorption coefficient, S is representing the dispersion coefficient, R is representing the color reflection and Ro is representing the uncolored (white) sample reflectance.

#### Colorfastness properties

The colorfastness to washing was determined using a Launder-Ometer following the AATCC test method 61-2013^29^. The colorfastness to rubbing (dry and wet) was determined using a Crockmeter following the AATCC test method 8-2016^30^. The colorfastness to perspiration (acid and alkaline) was determined by following the AATCC test method 15-2013^31^. The colorfastness to light was determined according to the AATCC test method 16.1–2014^32^. the grayscale reference for the color shift was used to evaluate washing, rubbing, and perspiration fastness properties, while the blue scale reference for color change was used to evaluate the light fastness property for dyed fabrics.

#### Dye fixation measurement

Dyed fabrics were washed at 50 °C for 20 min to test the dye fixation. The color strength values of the colored fabric after and before washing have been determined. The following equation was used to determine the dye fixation (%F)^21,33,34^.

% F = $$\:\frac{{(K/S)}_{a}}{{(K/S)}_{b}}\:\times\:100$$

where (K/S)_a_ is the color strength of the dyed fabric after washing and (K/S)_b_ is the color strength of the dyed fabric before washing.

#### RUI measurement

For the purpose of determining the reflectance values of ten randomly chosen locations on the dyed sample, a reflectance spectrophotometer operating in the visible spectrum region was utilised. An equation was used to determine the relative unlevelness index (RUI), where S represents the standard deviation (equation below), Rm represents the mean of reflectance values for n wavelengths, and V represents the photopic relative luminous efficiency function. The calculations were carried out using the equation below^21,34,35^. For each wavelength, Ri is the reflectance value of the measurement number i.

$$\:\text{S}{\uplambda\:}\:=\:\sqrt{\frac{{\sum\:}_{i=1}^{n}{({R}_{i}-\:{R}_{m})}^{2}}{n-1}}\:\text{R}\text{U}\text{I}\:=\:{\sum\:}_{\lambda\:=350}^{\lambda\:=700}(\frac{{S}_{\lambda\:}}{{R}_{m}}\times\:{V}_{\lambda\:})$$

It was determined that a levelness of 0.2 on the RUI was considered excellent, while a levelness of 0.49 was considered decent. The RUI values that fall between 0.5 and 1 are considered to be of poor levelness, whilst RUI values that are larger than 1 are considered to be of bad levelness^36^.

#### Mechanical properties

Dry crease recovery angle (CRA) was measured according to AATCC Test Method 66–2014^37^. Fabric roughness was measured using Surface Roughness measuring instrument SE 1700 a using ASTM Test Method D 7127–13^38^. Tensile strength and elongation at a break were measured using ASTM Test Method D5035-2011^39^. All reported values were the average of three readings.

#### Antimicrobial measurement

Nutrient agar broth media was prepared with pH 7.4 ± 0.2 according to the following recipe:

Yeast extract 2.0 g/L.

Peptone 5.0 g/L.

Meat extract 1.0 g/L.

NaCl 5.0 g/L.

Prepared media was stored below 8 °C, protected from direct light. Store dehydrated powder, in a dry place, in tightly sealed containers at 2–25 °C.

Bacteria used in this study were gram-negative bacteria: *Escherichia coli* (ATCC 25922), gram-positive bacteria: *Staphylococcus aureus* (ATCC 6538), and pathogenic yeast: *Candida albicans* (ATCC 10231). The inoculation of all microorganisms was prepared from fresh overnight broth cultures that were incubated at 37 °C^40^.

The inoculum size of these pathogenic strains was prepared and adjusted to approximately 0.5 McFarland standard (1.5 × 10^8^ CFU/mL), 25 µL of both bacterial & yeast suspensions were inoculated into each plate containing 20 mL of the sterile nutrient medium (NA).

The samples were applied to these tested microorganisms by using the shake flask method to calculate the antimicrobial activity throughout (%) reduction of the growth of these selected pathogenic strains was detected by optical density (OD) at 600.0 nm and the antimicrobial activity was measured throughout the relative [OD (%)] reduction of these pathogenic strains after treated with the textile disc samples compared to the control of these pathogenic strains have no any treatment^41–44^. All results were expressed according to the following equation:

$$\:Relative\:\left(OD\:Reduction\:\left(\text{\%}\right)\right)=\:\frac{A-B}{A}\:\times\:100$$

where A: the (OD) of the control flask contains pathogenic strains only without any treatment, B: the (OD) of tested flasks after applying a disc sample treated.

#### Adsorption experiments

A batch equilibrium approach was used to assess the adsorption capabilities of three different dye types [reactive, acid, and natural dye (black tea extract)] onto three different treated fabrics (cotton, wool, and cotton/wool blend) using two polycation polymers (chitosan and polyethyleneimine). In stopper flasks 1 g of treated fabric and 100 mL of different dye solutions (50–250 mg/L). The adsorption studies were carried out for 24 h at room temperature (25 °C) at 200 rpm on a thermostat shaker. UV–vis spectroscopy was used to determine the remaining concentration of dyes in the filtrate at a suitable wave number for each dye. To investigate the impact of adsorption isotherms, a series of isotherms were done at various pH= (4–10). Adsorption operations were carried out under ideal circumstances, with agitation times from 0 to 120 min. the amount of adsorbed dyes per gram [*q*_*e*_ (mg/g)] on the treated fabric (adsorption capacity) was determined using the following equation:^45^

$$\:{q}_{e}=\frac{\left({C}_{^\circ\:}-{C}_{e}\right)}{W}\:V\:$$

where *C*_*o*_ and *C*_*e*_ (mg/L) are the initial and equilibrium concentrations, respectively of the dye in solution, *V* (L) is the volume of dye solution and *W* (g) is the weight of the treated fabric.

## Results and discussion

### Characterization of the treated fabric using polycations

**Fig. 3:**
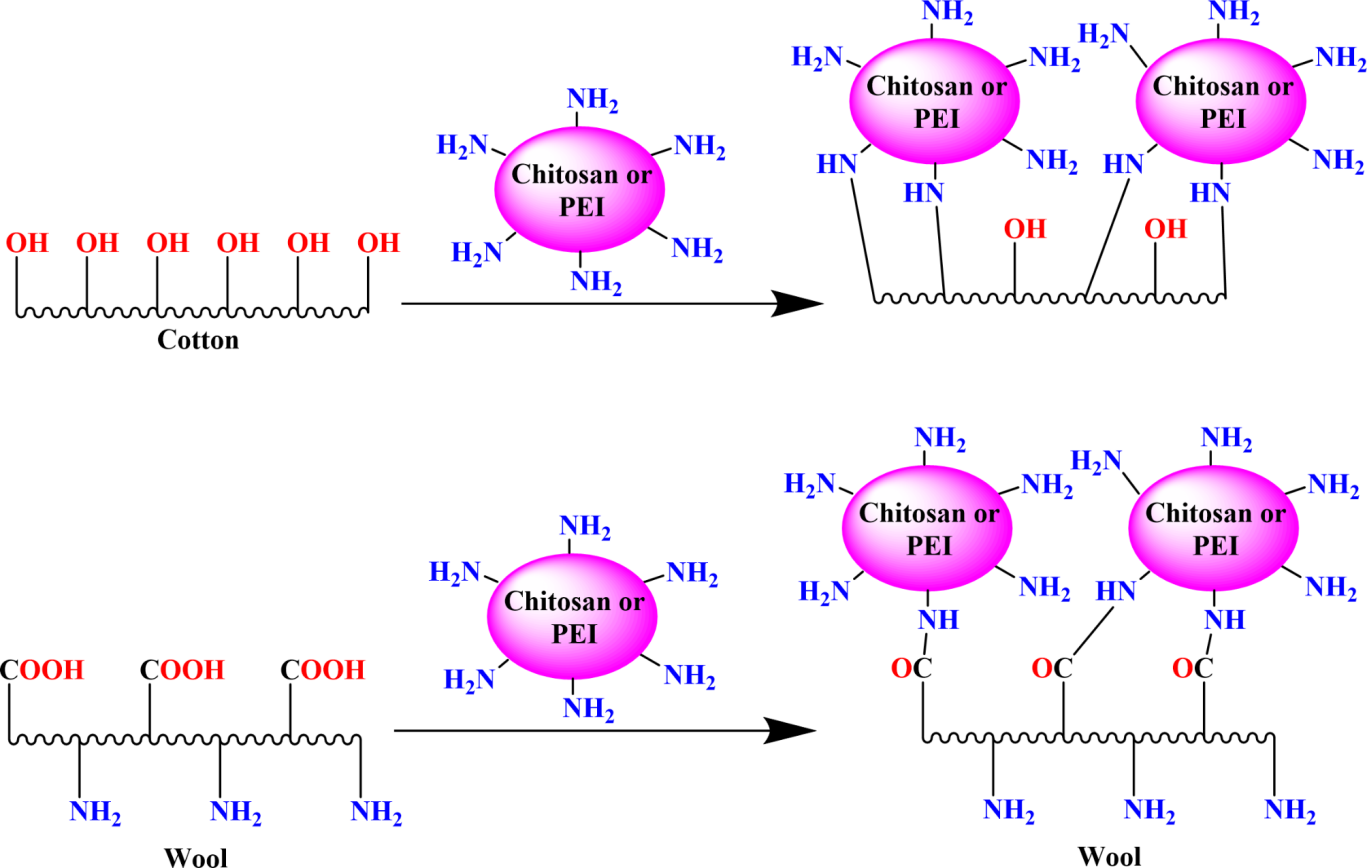
Mechanism of cotton and wool fabrics treated with different polycations.

Fabrics were soaked in the polymer for 20 min at 50 °C, then squeezed with 100% wet pickup and dried at 100 °C for 5 min before being dyed then cured at 140 °C for 5 min. Figure 3 depicts the response between fabric and polymeric materials.

In comparison to untreated textiles, pre-treated fabrics have a higher amine content when the polycations concentration is increased during the pre-treatment process. Furthermore, at all concentrations examined, pre-treated fabrics with PEI had a larger amine content than pre-treated fabric with chitosan (see Table 1). The important finding is that increasing the polycation concentration in the pre-treatment process by more than 1% results in a slight increase in the amine content values of treated fabrics, leading to conclude that, pre-treatment of fabrics with a 1% polycation compounds is more appropriate for this study.

Furthermore, the percent of amine content on the fabrics treated with PEI are higher than those treated with chitosan by comparing for the same concentration, this observation is attributed to the content of amine content in each treatment mater as the PEI have 25% from its weight primary amine while chitosan have lower value (12.4%) because some of its amino groups are presence as acetylated which lead to decrease the amount of amine content.

Additionally, the percentage of amine content on the fabrics treated with PEI is higher than that of the fabrics treated with chitosan when comparing the same concentration. This observation is attributed to the amine content in each treatment material, as PEI contains 25% of its weight as primary amine, whereas chitosan has a lower value of 12.4%. This is due to the fact that some of the amino groups in chitosan are acetylated, which results in a decrease in amine content.

**Fig. 4:**
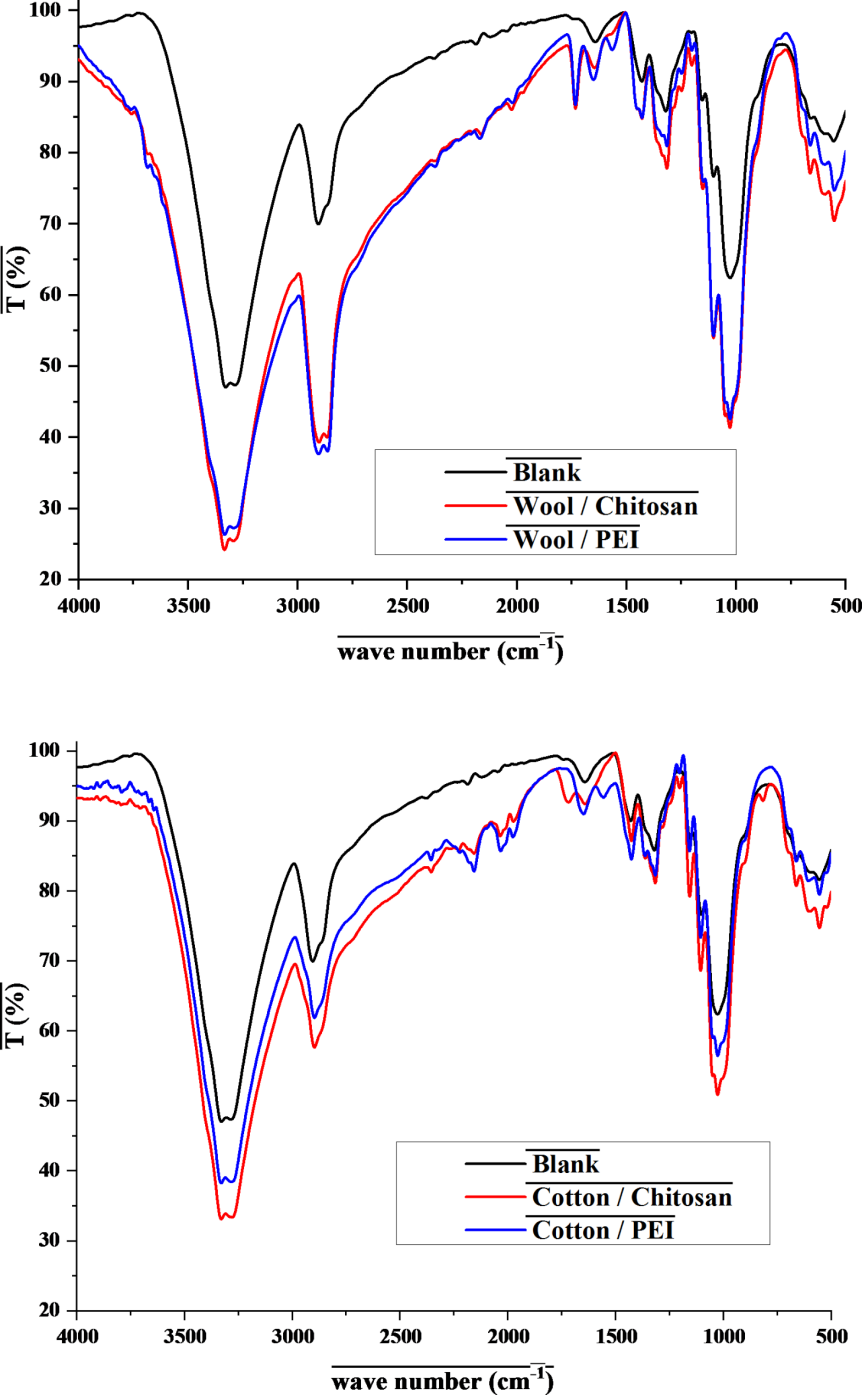
FT-IR spectra for treated fabrics with different composites.

Figure 4 shows the FTIR spectrum of the treated textiles. The amino groups of the polycations chain are responsible for the peak at 1650 cm^− 1^^46^. A shoulder peak occurred at 1722 cm^− 1^ when textile fabrics were modified with polycation compounds, indicating the formation of new amide groups as a result of the reaction between amine groups of polycations and hydroxyl or carboxyl groups of each fibers^47^.

Figure 4 shows the FTIR spectra of treated fabric with each polycation, which show that the NH_2_ group was presented in the 3000–3200 and 1590 cm^− 1^ absorption bands^48^. A stretch peak at 1245 cm^− 1^ has been discovered, which is connected to C-O from alcohol^49^. The ester or amide group has high wavelengths in the FTIR spectra, ranging from 1643 to 1657 cm^− 1^ which was the area for the C = O group^50^.

Tensile strength, elongation at break, roughness, and crease recovery angle in the warp and weft directions of cotton, wool, and cotton/wool fabrics were measured before and after they were treated with 1% polycations (PEI, and chitosan), and the findings are shown in Table 2.

Both chitosan and PEI have a positive effect on textile fabrics, as indicated by an increase in amine percent.

The results in Table 2 show that increasing the amine concentration in the treated textiles fabrics provides changes in the physico-mechanical characteristics, which were of particular interest. Fabric roughness, tensile strength, and elongation at the break and crease recovery angle all decrease by about (4–10%) for each fabric. This shows that the polycations under examination were deeply buried in the microstructure of the textile fabrics, forming a thin coating film on the fabric’s surface that was responsible for the observed modifications^51–54^.

**Table 1:** Amine content for treated fabrics using different polycation compounds.

Fabric	Polycation type	Polycation (%)	NH_2_ (mg/1 g fabric)	NH_2_%
Cotton	PEI	0	15.79 ± 0.97	1.21 ± 0.07
		0.5	307.96 ± 18.84	21.15 ± 1.29
		1	362.76 ± 22.2	42.31 ± 2.59
		2	381.03 ± 23.31	49.36 ± 3.02
		4	383.76 ± 23.48	54.21 ± 3.32
	Chitosan	0	15.79 ± 0.97	1.21 ± 0.07
		0.5	262.29 ± 16.05	13.3 ± 0.81
		1	271.42 ± 16.61	16.82 ± 1.03
		2	298.83 ± 18.28	27.39 ± 1.68
		4	307.32 ± 18.8	29.57 ± 1.81
Wool	PEI	0	19.14 ± 1.17	1.46 ± 0.09
		0.5	373.4 ± 22.85	25.65 ± 1.57
		1	439.86 ± 26.91	51.3 ± 3.14
		2	462 ± 28.27	59.85 ± 3.66
		4	465.31 ± 28.47	65.72 ± 4.02
	Chitosan	0	19.14 ± 1.17	1.46 ± 0.09
		0.5	318.02 ± 19.46	16.12 ± 0.99
		1	329.1 ± 20.14	20.4 ± 1.25
		2	362.33 ± 22.17	33.22 ± 2.03
		4	372.63 ± 22.8	35.85 ± 2.19
Cotton / Wool	PEI	0	17.46 ± 1.07	1.34 ± 0.08
		0.5	340.68 ± 20.84	23.4 ± 1.43
		1	401.31 ± 24.55	46.8 ± 2.86
		2	421.52 ± 25.79	54.6 ± 3.34
		4	424.54 ± 25.97	59.97 ± 3.67
	Chitosan	0	17.46 ± 1.07	1.34 ± 0.08
		0.5	290.16 ± 17.75	14.71 ± 0.9
		1	300.26 ± 18.37	18.61 ± 1.14
		2	330.58 ± 20.23	30.31 ± 1.85
		4	339.97 ± 20.8	32.7 ± 2

Treatment conditions: Polycations (X %); temperature: 50 °C, Time: 20 min, drying for 5 min at 100 °C.

**Table 2:** Amine content, physical and mechanical properties for treated fabrics using different polycation compounds.

Fabric	Polycation (1%)	NH_2_ (mg/1 g fabric)	NH_2_ (%)	Physical and Mechanical properties				
				R (µm)	Tensile strength (Kgf)	Elongation at a break (%)	CRA Warp (°)	CRA Weft (°)
Cotton	without	15.31 ± 1.88	1.17 ± 0.14	20.89 ± 2.56	153.89 ± 18.87	36.07 ± 4.42	120.72 ± 14.8	100.73 ± 12.35
	PEI	351.63 ± 43.12	41.01 ± 5.03	18.59 ± 2.28	137.8 ± 16.9	25.78 ± 3.16	113.02 ± 13.86	93.03 ± 11.41
	Chitosan	263.1 ± 32.27	16.31 ± 2	19.79 ± 2.43	140.3 ± 17.21	28.58 ± 3.5	103.93 ± 12.75	83.94 ± 10.29
Wool	without	18.56 ± 2.28	1.42 ± 0.17	21.39 ± 2.62	157.89 ± 19.36	36.97 ± 4.53	123.61 ± 15.16	103.63 ± 12.71
	PEI	426.36 ± 52.29	49.73 ± 6.1	19.09 ± 2.34	144.8 ± 17.76	26.38 ± 3.24	115.82 ± 14.2	95.83 ± 11.75
	Chitosan	319.01 ± 39.12	19.78 ± 2.43	20.39 ± 2.5	147.5 ± 18.09	29.28 ± 3.59	106.33 ± 13.04	86.34 ± 10.59
Cotton / Wool	without	16.93 ± 2.08	1.3 ± 0.16	21.98 ± 2.7	161.99 ± 19.87	37.97 ± 4.66	126.51 ± 15.52	106.53 ± 13.06
	PEI	389 ± 47.71	45.37 ± 5.56	19.49 ± 2.39	151.89 ± 18.63	27.08 ± 3.32	118.52 ± 14.53	98.53 ± 12.08
	Chitosan	291.05 ± 35.69	18.04 ± 2.21	20.89 ± 2.56	154.69 ± 18.97	30.08 ± 3.69	108.82 ± 13.35	88.84 ± 10.89

Treatment conditions: Polycations (1%); temperature: 50 °C, Time: 20 min, drying for 5 min at 100 °C.

R: Roughness, CRA: Crease Recovery Angle in warp and weft directions.

### Optimization of the dyeability parameters

#### Effect of polycation concentration

**Fig. 5:**
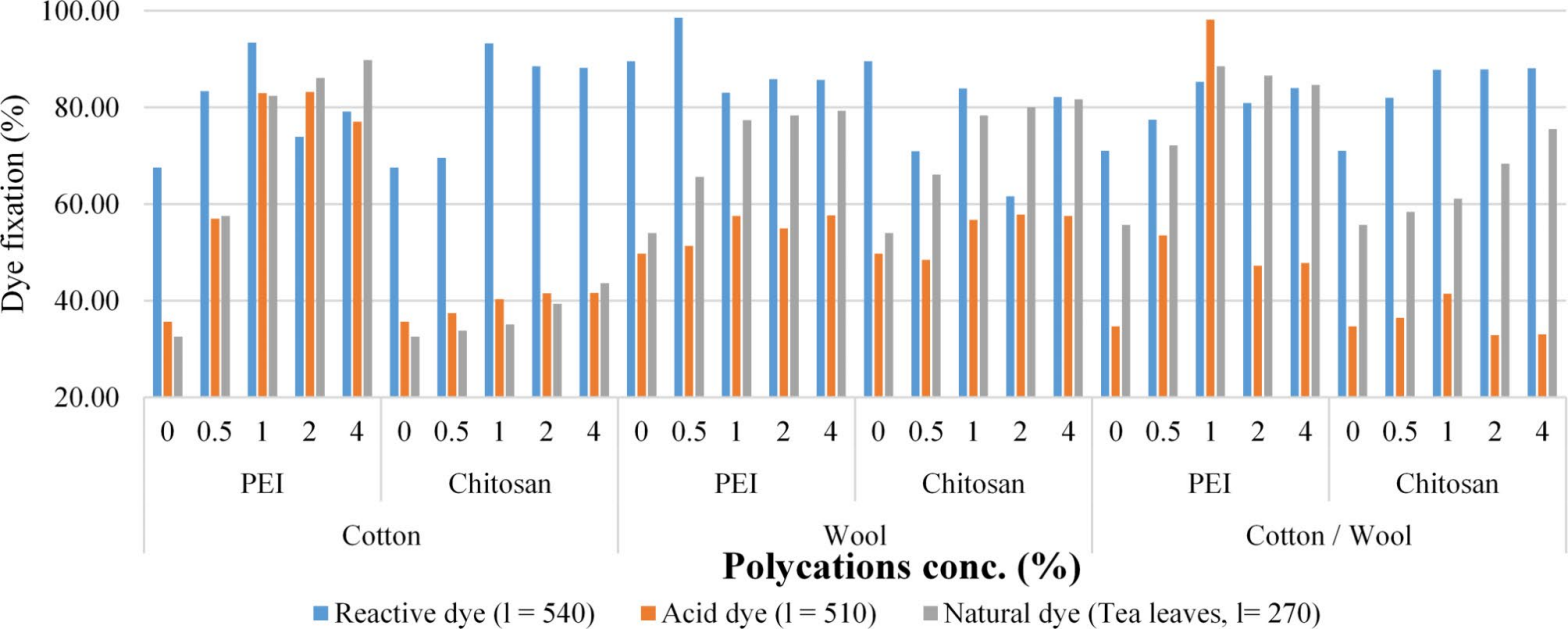
Color strength (K/S) values and dye fixation (%) for dyed treated fabrics with different concentrations of polycation compounds.

The amine content and color strength (K/S) values for dyed pre-treated fabrics with different concentrations of PEI and chitosan (0.5, 1, 2, and 4%) using different dyes (acid, reactive, and natural extracted dye from tea leaves) (for 20 min, at 50 °C) are shown in Table 3; Fig. 5.

When the concentration of polycation compounds in the pre-treatment procedure was increased, the amine content of the dyed fabric increased, which increased the color strength of the dyed fabric with all studied dyes (synthetic or natural) on all fabric types compared to the untreated one. Furthermore, at all concentrations examined, pre-treated fabric with PEI has a larger amine content and higher K/S values than pre-treated fabric with chitosan.

The main finding is that increasing the concentration of polycations in the pre-treatment process by more than 1% results in a minor increase in K/S values of dyed textiles, leading to the conclusion that pre-treatment of fabrics with a 1% polycations compound is more appropriate for this study. Looking for the fabric affinity to dye, it is clear that, both cotton or wool fabrics or their blend provide a good color strength and dye fixation upon treatment with polycation compounds.

According to the equation described in the experimental section, the dye fixation of dyed untreated and treated textiles was computed. Treatment with polycation compounds enhanced the dye-fixing of employed dyes (reactive, acid, and/or natural dye) on all materials, as shown in Table 3. This rise was greater in PEI-treated textiles than in chitosan-treated fabrics. The chemical interactions between (i) amino groups from polycation compounds, (ii) hydroxyl groups on the cotton surface or carboxyl groups from the wool surface, and (iii) dye molecules have improved the dye-fixing significantly when both polycations were utilized.

**Table 3:** Amine content and color strength (K/S) values for dyed treated fabrics using different polycation compounds.

Fabric	Polycation type	Polycation (%)	NH2 (mg/1 g fabric)	NH_2_%	Color Performance								
					Reactive dye (λ = 540 nm)			Acid dye (λ = 510 nm)			Natural dye (Tea leaves, λ = 270 nm)		
					before washing	after washing	Dye fixation (%)	before washing	after washing	Dye fixation (%)	before washing	aftre washing	Dye fixation
Cotton	PEI	0	15.79 ± 0.97	1.21 ± 0.07	3.55	2.40	67.57	3.11	1.11	35.67	10.16	3.31	32.58
		0.5	307.96 ± 18.84	21.15 ± 1.29	11.33	9.45	83.41	9.97	5.68	56.97	15.80	9.09	57.50
		1	362.76 ± 22.2	42.31 ± 2.59	12.79	11.95	93.43	8.04	6.67	82.99	18.03	14.86	82.42
		2	381.03 ± 23.31	49.36 ± 3.02	13.36	9.87	73.88	7.43	6.18	83.21	17.93	15.44	86.11
		4	383.76 ± 23.48	54.21 ± 3.32	12.45	9.85	79.12	8.09	6.23	77.04	17.83	16.01	89.79
	Chitosan	0	15.79 ± 0.97	1.21 ± 0.07	3.55	2.40	67.57	3.11	1.11	35.67	10.16	3.31	32.58
		0.5	262.29 ± 16.05	13.3 ± 0.81	3.52	2.45	69.60	3.07	1.15	37.45	11.67	3.95	33.84
		1	271.42 ± 16.61	16.82 ± 1.03	3.28	3.06	93.29	3.13	1.26	40.32	13.08	4.59	35.09
		2	298.83 ± 18.28	27.39 ± 1.68	3.31	2.93	88.52	2.91	1.21	41.53	12.27	4.83	39.38
		4	307.32 ± 18.8	29.57 ± 1.81	4.16	3.67	88.22	2.91	1.21	41.62	11.61	5.07	43.67
Wool	PEI	0	19.14 ± 1.17	1.46 ± 0.09	0.77	0.69	89.61	8.14	4.05	49.75	8.15	4.40	53.99
		0.5	373.4 ± 22.85	25.65 ± 1.57	6.19	6.10	98.55	8.88	4.56	51.35	11.38	7.48	65.67
		1	439.86 ± 26.91	51.3 ± 3.14	7.68	6.38	83.07	8.48	4.88	57.57	13.64	10.55	77.35
		2	462 ± 28.27	59.85 ± 3.66	8.20	7.04	85.89	8.90	4.89	54.93	14.59	11.43	78.32
		4	465.31 ± 28.47	65.72 ± 4.02	8.19	7.02	85.71	8.38	4.83	57.66	15.53	12.31	79.29
	Chitosan	0	19.14 ± 1.17	1.46 ± 0.09	0.77	0.69	89.61	8.14	4.05	49.75	8.15	4.40	53.99
		0.5	318.02 ± 19.46	16.12 ± 0.99	3.85	2.73	70.91	9.88	4.79	48.46	8.71	5.76	66.16
		1	329.1 ± 20.14	20.4 ± 1.25	4.62	3.88	83.98	8.71	4.94	56.70	9.09	7.12	78.33
		2	362.33 ± 22.17	33.22 ± 2.03	4.64	2.86	61.64	8.38	4.85	57.86	8.80	7.04	80.02
		4	372.63 ± 22.8	35.85 ± 2.19	2.69	2.21	82.16	8.43	4.85	57.53	8.52	6.96	81.70
Cotton / Wool	PEI	0	17.46 ± 1.07	1.34 ± 0.08	3.57	2.54	71.05	2.68	0.93	34.70	10.07	5.61	55.71
		0.5	340.68 ± 20.84	23.4 ± 1.43	11.67	9.04	77.48	8.09	4.33	53.52	10.80	7.79	72.13
		1	401.31 ± 24.55	46.8 ± 2.86	12.25	10.45	85.31	5.03	4.94	98.21	11.26	9.97	88.54
		2	421.52 ± 25.79	54.6 ± 3.34	11.17	9.04	80.92	7.28	3.44	47.25	13.17	11.41	86.59
		4	424.54 ± 25.97	59.97 ± 3.67	11.62	9.77	84.06	7.40	3.54	47.84	15.17	12.84	84.64
	Chitosan	0	17.46 ± 1.07	1.34 ± 0.08	3.57	2.54	71.05	2.68	0.93	34.70	10.07	5.61	55.71
		0.5	290.16 ± 17.75	14.71 ± 0.9	3.15	2.58	82.02	3.07	1.12	36.45	10.70	6.25	58.43
		1	300.26 ± 18.37	18.61 ± 1.14	2.96	2.60	87.84	3.18	1.32	41.46	11.27	6.89	61.14
		2	330.58 ± 20.23	30.31 ± 1.85	3.25	2.86	87.91	3.92	1.29	32.90	11.76	8.04	68.35
		4	339.97 ± 20.8	32.7 ± 2	3.38	2.98	88.14	3.87	1.28	33.06	12.15	9.18	75.56

Treatment conditions: Polycations (X %), temperature: 50 °C, Time: 20 min, drying for 5 min at 100 °C.

Dyeing conditions: dye concentration (0.5 g/L); Polycations (X %); dyeing temperature: 60 °C, Dyeing Time: 20 min, drying for 5 min at 100 °C, curing for 5 min at 140°C Effect of Dyeing Temperature.

**Fig. 6:**
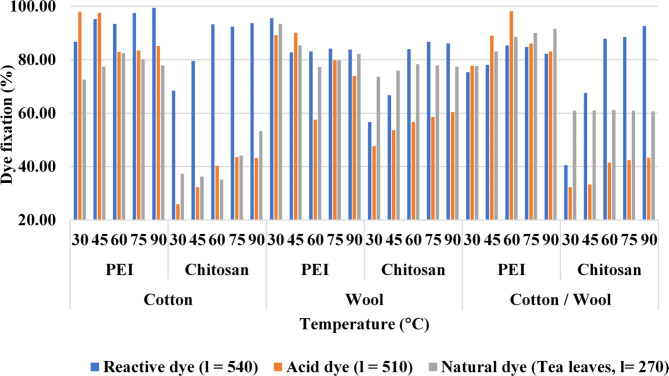
Color strength (K/S) values and dye fixation (%) for dyed treated fabrics at different dyeing temperatures.

Table 4; Fig. 6 shows the color strength (K/S) values for dyed-treated textile fabrics with polycations (1%) at various dyeing temperatures (30, 45, 60, 75, and 90 °C). The color strength of fabrics increases as the dyeing temperature rises from 30 to 75 °C. Because of this, increasing the temperatures causes fiber swelling and destroys dye molecule agglomeration, which improves dye penetration into the modified fabrics^55–57^.

As demonstrated in Table 4, treatment with polycation compounds improved the dye-fixing of the utilized dyes which are reactive dye (Synozol RED K 3BS), acid dye (Acid red 27), and/or natural dye (tea leaves extract) on all substrates upon increasing the dyeing temperature from 30 to 75 °C and further increasing in the dyeing temperature causing slight changes in the color fastness and dye fixation. PEI-treated textiles had a higher increase than chitosan-treated textiles by about (200%) as PEI provides more amine content which provides more interactions between dyes molecules and fabric surface.

Another observation, reactive dye provides a higher dye fixation percent than other examined dyes (acid or natural). In addition, increasing the temperature provides high color strength for fabrics dyed with natural dye.

**Table 4:**
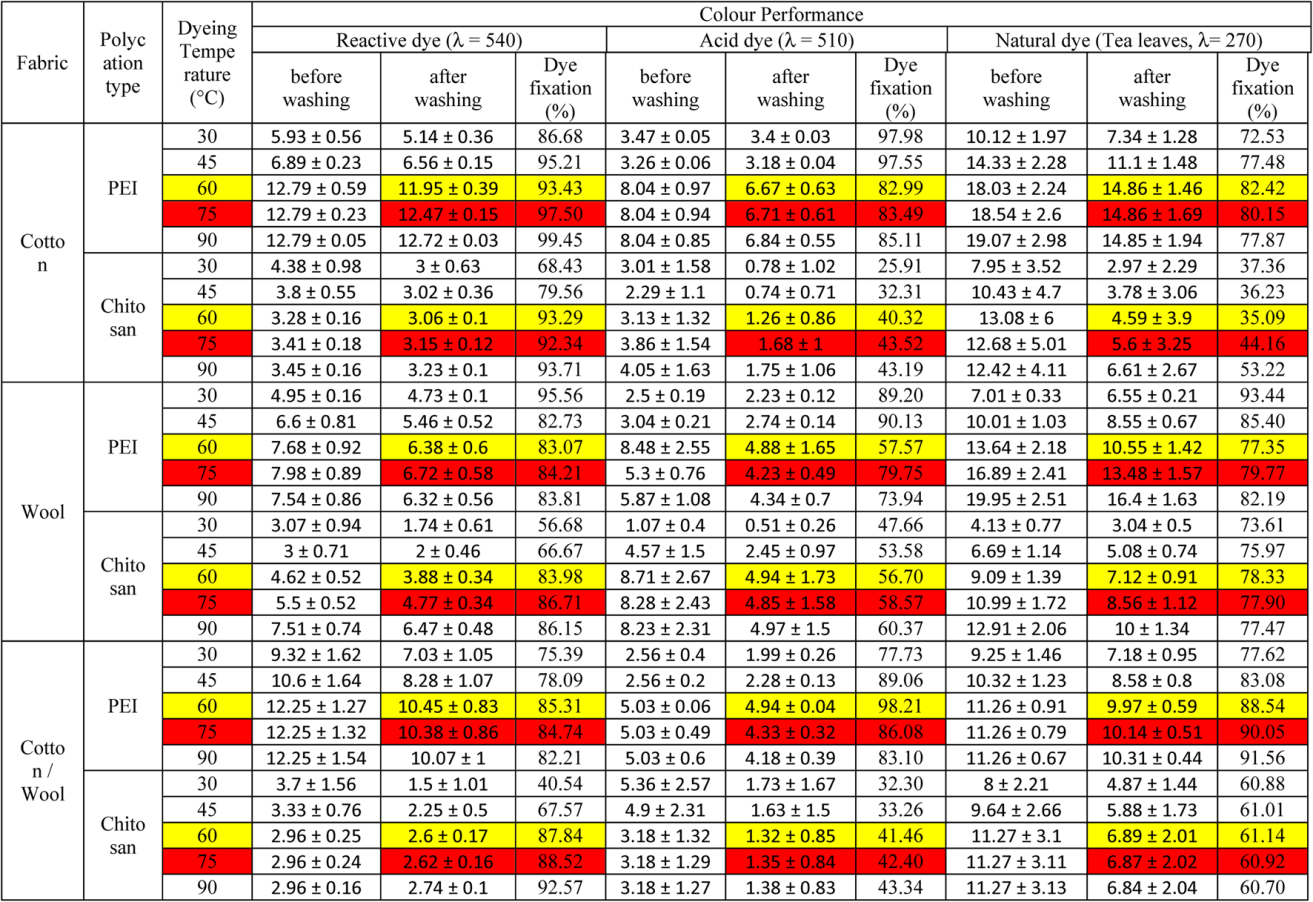
Effect of Dyeing temperature on the color performance of treated fabrics.

#### Effect of dyeing time

**Fig. 7:**
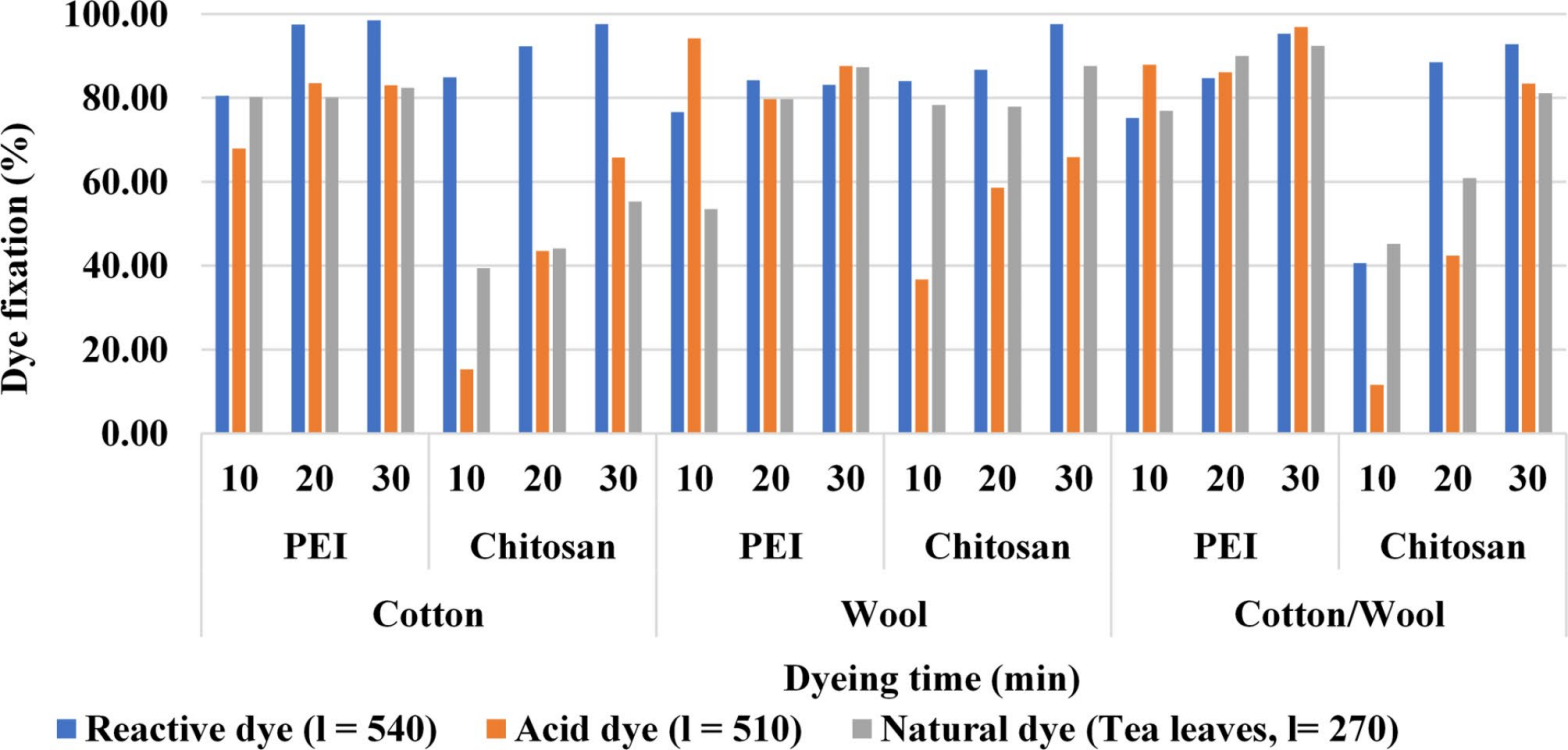
Color strength (K/S) values and dye fixation (%) for dyed treated fabrics at different dyeing time.

Table 5; Fig. 7 shows how the color intensity of dyed treated fabrics with all examined dyes which are reactive dye (Synozol RED K 3BS), acid dye (Acid red 27), and/or natural dye (tea leaves extract) has increased as the dyeing time increased from 10 to 20 min (after 75 °C was reatched). The color intensity was slightly increased as the dyeing time increased to 30 min. The coloring process seemed to have reached equilibrium after 20 min. Heating disturbs the equilibrium over time, resulting in a slight change in the K/S ratio^58^. The partial breakdown of dye molecules caused by extended contact with high temperatures might account for this decline^55^.

Polycation compounds play an important role in this investigation as at a suitable temperature of 75 °C, all amine groups are highly active so they absorb and react with a fabric surface and dye molecule, and increasing the time provides coating another dyes layer which leaking from the fabric surface upon washing. This was confirmed by the dye fixation values.

**Table 5:**
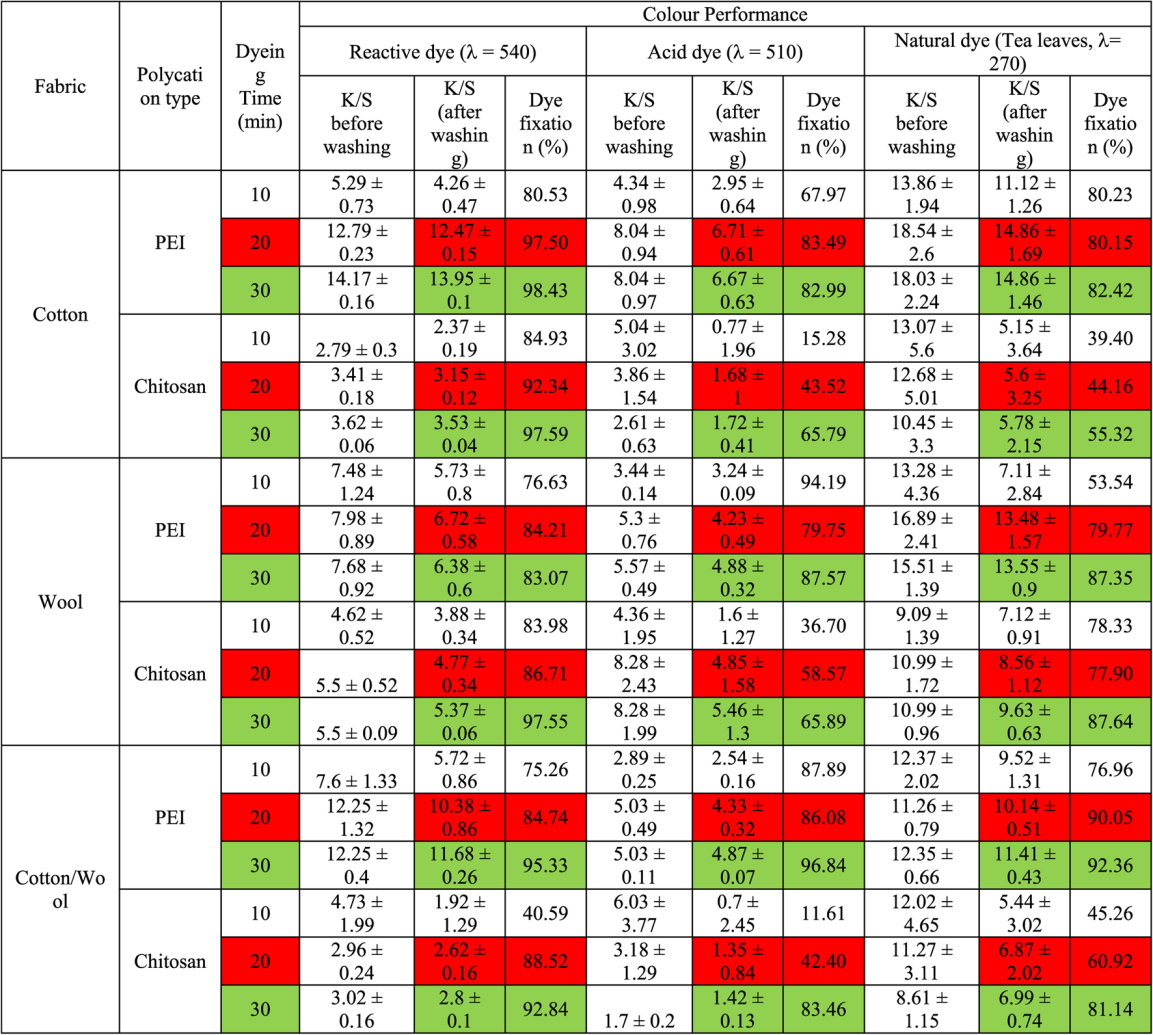
Effect of dyeing time on the color performance of treated fabrics.

#### Characterization of the dyed textile fabrics

##### Fastness properties

Fastness properties for the dyed treated fabrics (cotton, wool, and cotton/wool) with a polycation (PEI and chitosan; 1%) using three different dye natures which are reactive dye (Synozol RED K 3BS), acid dye (Acid red 27) and/or natural dye (tea leaves extract) at optimum finding dyeing condition were shown in Table 6.

When compared to untreated materials, treated fabrics had better washing as it enhanced from (2–3) to (4–5) for all types of treated fabrics with all types of dyes, perspiration as it enhanced from (2–3) to (4–5) for all types of treated fabrics with all types of dyes, lighting as it enhanced from (2–3) to (4–5) for all types of treated fabrics with all types of dyes, and rubbing fastness properties as it enhanced from (2–3) to (4–5) for all types of treated fabrics with all types of dyes. In comparison to untreated materials, chitosan and polyethyleneimine treatment considerably improved the washing and light fastness characteristics of colored fabrics using all studied dyes, since the polycation treated fabrics absorb more color molecules. The light-fastness properties of modified textiles improved as the dye level in the fiber increased. This improvement is due to the chemical linkages formed between polycations-treated materials and dye molecules.

Furthermore, increased hydrogen bonding between dye molecules, polycations, and the fabric surface is responsible for the dye molecules being fixed within and on the textile surface.

In addition, when the fabric is treated with a polycation, the dyeing homogeneity is improved because the fibers have a uniform thin coating of polycations on the surface, and the dye molecules are absorbed better and more consistently.

After curing step, most of the adsorbed dyes was fixed on the fabric surface inside the polymer network, and after washing step the unreacted dyes were throughout (the dyes was adsorbed in multilayers form). So the fixed dyes on the treated fabrics was permanent chemically reacted and provide good fastness properties as the polymer network serve the dyes from leaking.

**Table 6:** Fastness properties for the dyed treated fabrics with polycations using different dyes nature.

Fabric	Dye	Polycation (1%)	Fastness properties											RUI	
			Washing		Rubbing				Perspiration				Light		
					Dry		Wet		Acidic		Alkaline				
			Alt	St	Alt	St	Alt	St	Alt	St	Alt	St		value	rate
Cotton	Reactive dye	without	3	2–3	3	2–3	3	2–3	3	2–3	3	2–3	4	0.541	Poor
		PEI	5	5	4–5	4–5	4–5	4–5	4	4–5	4–5	4–5	5	0.188	Excellent
		Chitosan	4–5	4	3–4	4	4	4	4	4	4	4	5	0.413	Good
	Acid dye	without	2	2	2	2	2–3	2–3	2	2	2	2	3–4	0.587	Poor
		PEI	5	5	5	5	4–5	4–5	4–5	5	5	5	4–5	0.226	Good
		Chitosan	4–5	4	4	4–5	4–5	4	4	5	4	4	4	0.355	Good
	Natural dye	without	2	2	2	2	2	2	2	2	2	2	2–3	0.535	Poor
		PEI	4	4	3–4	4	4	3–4	4	4	4	4	4	0.222	Good
		Chitosan	3–4	4	3	3–4	3–4	3	4	4	4	4	4	0.390	Good
Wool	Reactive dye	without	3–4	3	3	3–4	3–4	3	3	3	3	3	4	0.532	Poor
		PEI	4–5	5	3–4	4–5	4–5	4–5	4–5	5	5	5	4–5	0.227	Good
		Chitosan	4–5	4–5	4–5	4–5	4–5	4–5	4–5	4–5	4–5	4–5	4–5	0.319	Good
	Acid dye	without	3	3	3	3	3	3	3	3	3	3	3–4	0.523	Poor
		PEI	5	5	4–5	4–5	4–5	4–5	4–5	5	5	5	4–5	0.319	Good
		Chitosan	4	4	4	4–5	4	4–5	4	4–5	4	4	4–5	0.353	Good
	Natural dye	without	3	2–3	3	2–3	3	2–3	3	2–3	3	2–3	4	0.541	Poor
		PEI	4–5	4	4–5	3–5	4–5	5	4–5	4–5	4–5	4–5	5	0.294	Good
		Chitosan	4	3–4	4–5	5	4–5	4–5	4–4	4–5	4	4	5	0.333	Good
Cotton / Wool	Reactive dye	without	3	3	4	3–4	3	3	3	3	3	3	3	0.482	Good
		PEI	5	5	4–5	4–5	4–5	4–5	4–5	5	5	5	4–5	0.293	Good
		Chitosan	4–5	4	4	4–5	4–5	4	4	4	4	4	4	0.442	Good
	Acid dye	without	2–3	2–3	3	2–3	3	2–3	3	2–3	3	2–3	4	0.403	Good
		PEI	5	4–5	4–5	5	4–5	4–5	4–5	4–5	4	4	5	0.281	Good
		Chitosan	3–4	4	4	4–5	4–5	4	4	4	4	4	4	0.359	Good
	Natural dye	without	2–3	2–3	3	3	3	2–3	3	3	3	3	3	0.456	Good
		PEI	4–5	4	4	4–5	4–5	4–5	4–5	5	5	4	4–5	0.209	Good
		Chitosan	3	3–4	3–4	3–4	4	3–4	4	4–5	4	3	4–5	0.318	Good

Treatment condition: polymer conc. 1%, temperature: 50 °C, Time: 20 min, drying at 100 °C for 5 min.

Dyeing condition: Dye concentration 0.5 g/kg (for reactive and acid dyes) and 100% (natural dye extract), Dyeing temperature: 75 °C, dyeing time: 20 min, drying at 100 °C for 5 min. and curing for 140 °C for 5 min.

Washing, rubbing and perspiration fastness properties scale from (1–5): 1 means bad, 2 means poor, 3 means good, 4 means very good, 5 means excellent.

Light fastness scale from (1–8:^1,2^ means poor,^3,4^ means good,^5,6^ means very good,^7,8^ means excellent.

RUI values range can be categorized into If RUI < 0.2 that considered as Excellent levelness. If 0.2 < RUI < 0.49 that considered as Good levelness, If RUI is between 0.5 and 1 that means Poor levelness. If RUI values are greater than 1 that indicate Bad levelness.

#### Antimicrobial activity

In Table 7, antimicrobial actions of cotton, wool, and cotton/wool fabrics treated with 1% polycations and dyed with three different dye natures (Synozol RED K 3BS), acid dye (Acid red 27) and/or natural dye (tea leaves extract) were demonstrated using three types of microorganisms (i) gram-positive (Staphylococcus Aureus), (ii) gram-negative (Escherichia coli), and (iii) fungal (Candida Albicans).

The findings demonstrate that two kinds of bacteria (gram-positive and negative bacteria), as well as a fungus (Candida Albicans), tested on untreated dyed textile materials (blank), had an inhibitory impact better than on blank material without dyeing. The presence of phenolic groups in reactive or acid dyes, as well as phenolic acids and flavonoids in natural dye extract, is responsible for this activity.

Because both the examined bacterial strains have different compositional variances in cell walls, the treated textiles are more effective with gram-negative bacteria than gram-positive bacteria. Ergosterol, a key component of the fungal cell membrane, is likewise inhibited by the polycations in use^27,59–64^.

PEI-treated textiles have a greater antibacterial effect than chitosan-treated fabrics, due to the presence of a higher percent of amino groups, which play an important role in damaging microbial cell membranes and providing a powerful antibacterial effect^13^. Metals, phenolic acids, and flavonoids found in natural dye extract (tea extract) have successfully interacted with the bacterium’s cells. The polycation chemicals contained in the cover disperse and stabilize the extract dyes components (Metals, phenolic acids, and flavonoids) well on the fabric surface, increasing their ability to interact with bacterial cells on the fabric surface^48,65–67^.

Furthermore, treated and dyed textile materials have better antibacterial properties than untreated textile fabrics before and after washing. After washing, the durability of the treated fabrics provides an excellent antibacterial effect against all investigated microorganisms.

**Table 7:** CFU reduction (%) of microbial strain cells for untreated and treated dyed fabrics before and after washing.

Fabric	Dye Type	Polycation (1%)	Microbial Reduction %					
			E. coli (ATCC 25922)		S. Aureus (ATCC 29213)		C. Albicans (ATCC 10231)	
			before washing	after 10 washing cycles	before washing	after 10 washing cycles	before washing	after 10 washing cycles
Cotton	reactive dye	without	26.1 ± 3.96	20.5 ± 2.57	28.3 ± 5.8	20.1 ± 3.77	27.1 ± 6.22	18.3 ± 4.04
		PEI	88.5 ± 9.76	74.7 ± 6.34	95.9 ± 16.05	73.2 ± 10.43	92 ± 18.03	66.5 ± 11.72
		Chitosan	78.3 ± 8.63	66.1 ± 5.61	84.8 ± 14.14	64.8 ± 9.19	81.4 ± 15.98	58.8 ± 10.39
	acid dye	without	45.6 ± 6.93	35.8 ± 4.5	49.4 ± 10.11	35.1 ± 6.57	47.3 ± 10.89	31.9 ± 7.08
		PEI	94 ± 6.15	85.3 ± 4	101.8 ± 12.87	83.6 ± 8.37	97.6 ± 15.34	75.9 ± 9.97
		Chitosan	82.4 ± 5.73	74.3 ± 3.72	86.2 ± 9.48	72.8 ± 6.16	82.7 ± 10.75	67.5 ± 6.99
	natural dye	without	65 ± 9.83	51.1 ± 6.39	70.4 ± 14.35	50.1 ± 9.33	67.5 ± 15.56	45.5 ± 10.11
		PEI	99.4 ± 2.47	95.9 ± 1.61	107.7 ± 9.76	93.9 ± 6.34	103.2 ± 12.66	85.3 ± 8.23
		Chitosan	86.4 ± 2.76	82.5 ± 1.79	87.5 ± 4.74	80.8 ± 3.08	83.9 ± 5.44	76.2 ± 3.54
Wool	reactive dye	without	41 ± 3.32	36.3 ± 2.16	44.4 ± 6.22	35.6 ± 4.04	42.6 ± 7.28	32.3 ± 4.73
		PEI	89.4 ± 3.04	85.1 ± 1.98	96.9 ± 9.55	83.4 ± 6.2	92.9 ± 12.16	75.7 ± 7.91
		Chitosan	82.5 ± 5.94	74.1 ± 3.86	89.4 ± 11.88	72.6 ± 7.72	85.7 ± 14	65.9 ± 9.1
	acid dye	without	55.6 ± 4.45	49.3 ± 2.9	60.2 ± 8.41	48.3 ± 5.47	57.7 ± 9.83	43.8 ± 6.39
		PEI	91.7 ± 3.11	87.3 ± 2.02	96.5 ± 7.78	85.5 ± 5.06	95.3 ± 12.52	77.6 ± 8.14
		Chitosan	85.9 ± 6.22	77.1 ± 4.04	89.6 ± 9.97	75.5 ± 6.48	89.2 ± 14.57	68.6 ± 9.47
	natural dye	without	70.2 ± 5.66	62.2 ± 3.68	76 ± 10.61	61 ± 6.89	72.9 ± 12.45	55.3 ± 8.09
		PEI	94.1 ± 3.25	89.5 ± 2.11	96.2 ± 6.01	87.7 ± 3.91	97.7 ± 12.8	79.6 ± 8.32
		Chitosan	89.2 ± 6.43	80.1 ± 4.18	89.7 ± 7.92	78.5 ± 5.15	92.7 ± 15.2	71.2 ± 9.88
Cotton / Wool	reactive dye	without	35.8 ± 0.35	36.3 ± 0.23	38.7 ± 2.19	35.6 ± 1.42	37.1 ± 3.39	32.3 ± 2.21
		PEI	82.4 ± 0.92	83.7 ± 0.6	89.2 ± 5.09	82 ± 3.31	85.6 ± 7.92	74.4 ± 5.15
		Chitosan	76.3 ± 0.92	77.6 ± 0.6	82.7 ± 4.74	76 ± 3.08	79.3 ± 7.28	69 ± 4.73
	acid dye	without	57.2 ± 0.64	58.1 ± 0.41	62 ± 3.61	56.9 ± 2.34	59.4 ± 5.44	51.7 ± 3.54
		PEI	87.7 ± 0.99	89.1 ± 0.64	92.2 ± 3.46	87.3 ± 2.25	91.1 ± 8.41	79.2 ± 5.47
		Chitosan	83.1 ± 0.92	84.4 ± 0.6	86.5 ± 2.69	82.7 ± 1.75	86.3 ± 7.92	75.1 ± 5.15
	natural dye	without	78.7 ± 0.85	79.9 ± 0.55	85.2 ± 4.88	78.3 ± 3.17	81.7 ± 7.5	71.1 ± 4.87
		PEI	93 ± 1.06	94.5 ± 0.69	95.1 ± 1.84	92.5 ± 1.2	96.6 ± 8.91	84 ± 5.79
		Chitosan	89.9 ± 0.99	91.3 ± 0.64	90.4 ± 0.71	89.4 ± 0.46	93.4 ± 8.63	81.2 ± 5.61

Treatment condition: polymer conc. 1%, temperature: 50 °C, Time: 20 min, drying at 100 °C for 5 min.

Dyeing condition: Dye concentration 0.5 g/kg (for reactive and acid dyes) and 100% (natural dye extract), Dyeing temperature: 75 °C, dyeing time: 20 min, drying at 100 °C for 5 min. and curing for 140 °C for 5 min.

Washing, rubbing and perspiration fastness properties scale from (1–5): 1 means bad, 2 means poor, 3 means good, 4 means very good, 5 means excellent.

Light fastness scale from (1–8:^1,2^ means poor,^3,4^ means good,^5,6^ means very good,^7,8^ means excellent.

### Dye adsorption

The dye adsorption capability of the treated fabrics was studied using three dyes with different natures (Synozol RED K 3BS), acid dye (Acid red 27), and/or natural dye (tea leaves extract). Initial contact duration and various kinetic and isothermal factors were all studied, to see how they affected dye adsorption onto different fabrics (cotton, wool, and cotton/wool blend).

#### Effect of pH on adsorption

The adsorption process is influenced by the pH of the dye solution, which influences the surface charge of the fabrics and the protonation degree of the functional groups^68^. At an appropriate starting concentration of different dyes, the influence of pH on dye adsorption capability by treated fabrics was investigated. Diluted NaOH and HCl solution was used to change the pH between 4 and 10.

**Fig. 8:**
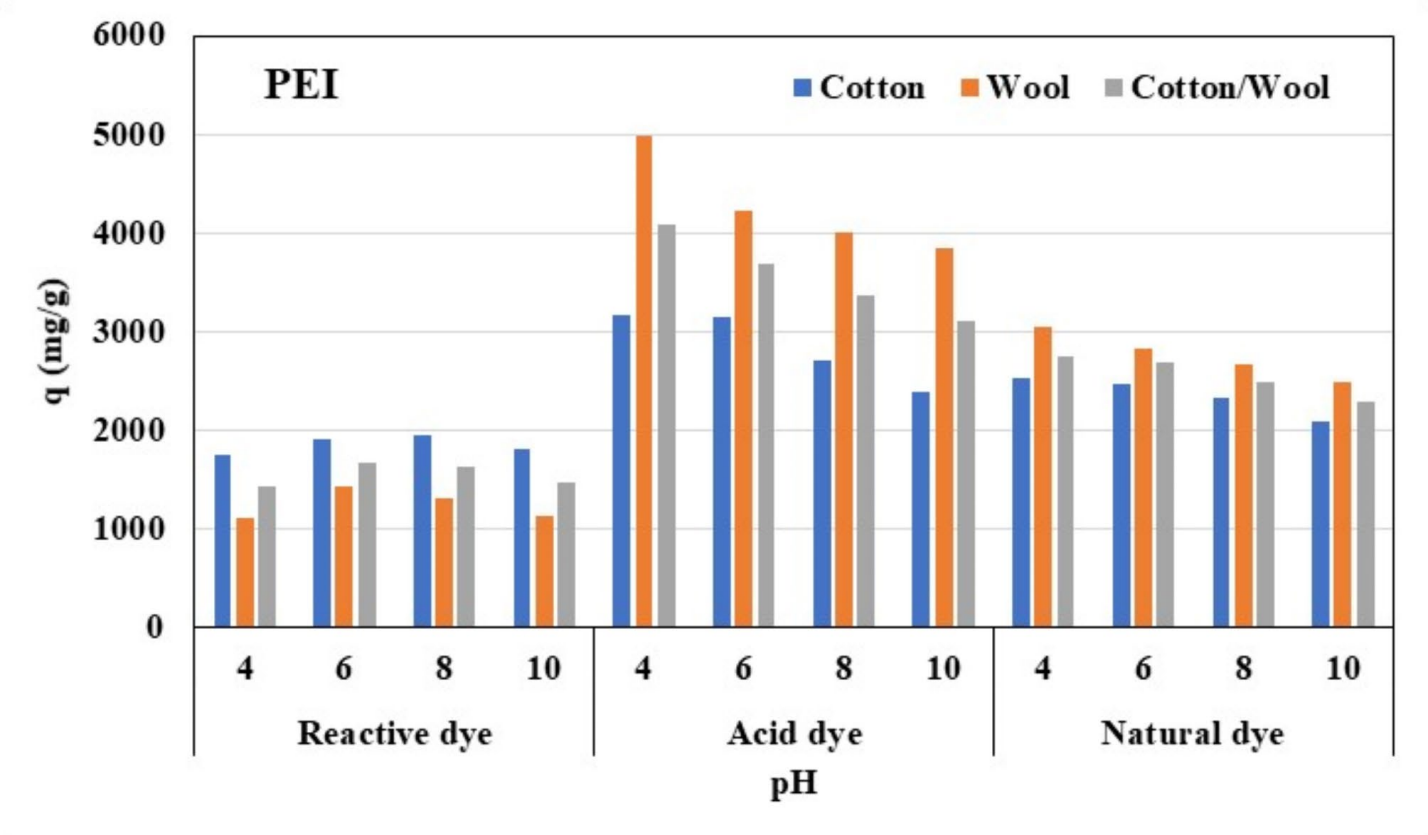
Effect of pH on the adsorption of different dyes nature onto different treated fabrics with PEI.

**Fig. 9:**
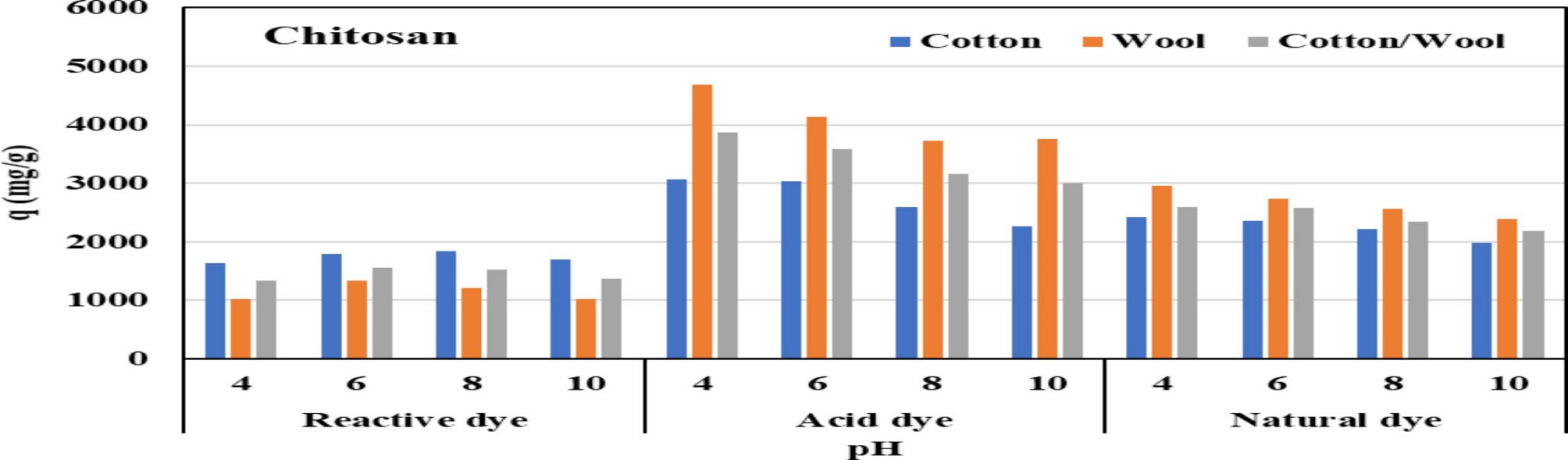
Effect of pH on the adsorption of different dyes nature onto different treated fabrics with chitosan.

Figures 8 and 9 shows the pH influence on the adsorption capabilities of three investigated dyes using three different treated fabrics using chitosan and polyethyleneimine at room temperature (25 °C). For reactive dye, it is clear that, when pH values rise, the adsorption capacity of investigated dyes increases at first, then declines. When the pH reaches 7, the maximal adsorption capacity is reached. The amino groups on the treated fabrics were the best groups for forming hydrogen bonds between the dyes and the fabrics when the pH increased from 4 to 8. When the pH of the dye solution is higher than 10, the cationic group repulsion that is created on the surface of the fabric causes the adsorption capability of dyes to decrease^69^.

It is also noted that, for acid and natural dye, the maximum adsorption capacity is reached at pH 4 and when the pH increases up to pH 10 the maximum adsorption capacity was decreased. the pH is lower than the pKa of the amino group (9.6). This means that the amino group remains protonated. And if the pH of solution greater than 9.6, This means that amino group now deprotonated. and negatively charged. as acid dye and natural dyes have hydroxyl group in their structure and the cationic fabric have highly protonated NH2 group at lower pH therefore the maximum capacity could be at lower pH than our investigated pH (4).

#### Effect of time on adsorption

**Fig. 10:**
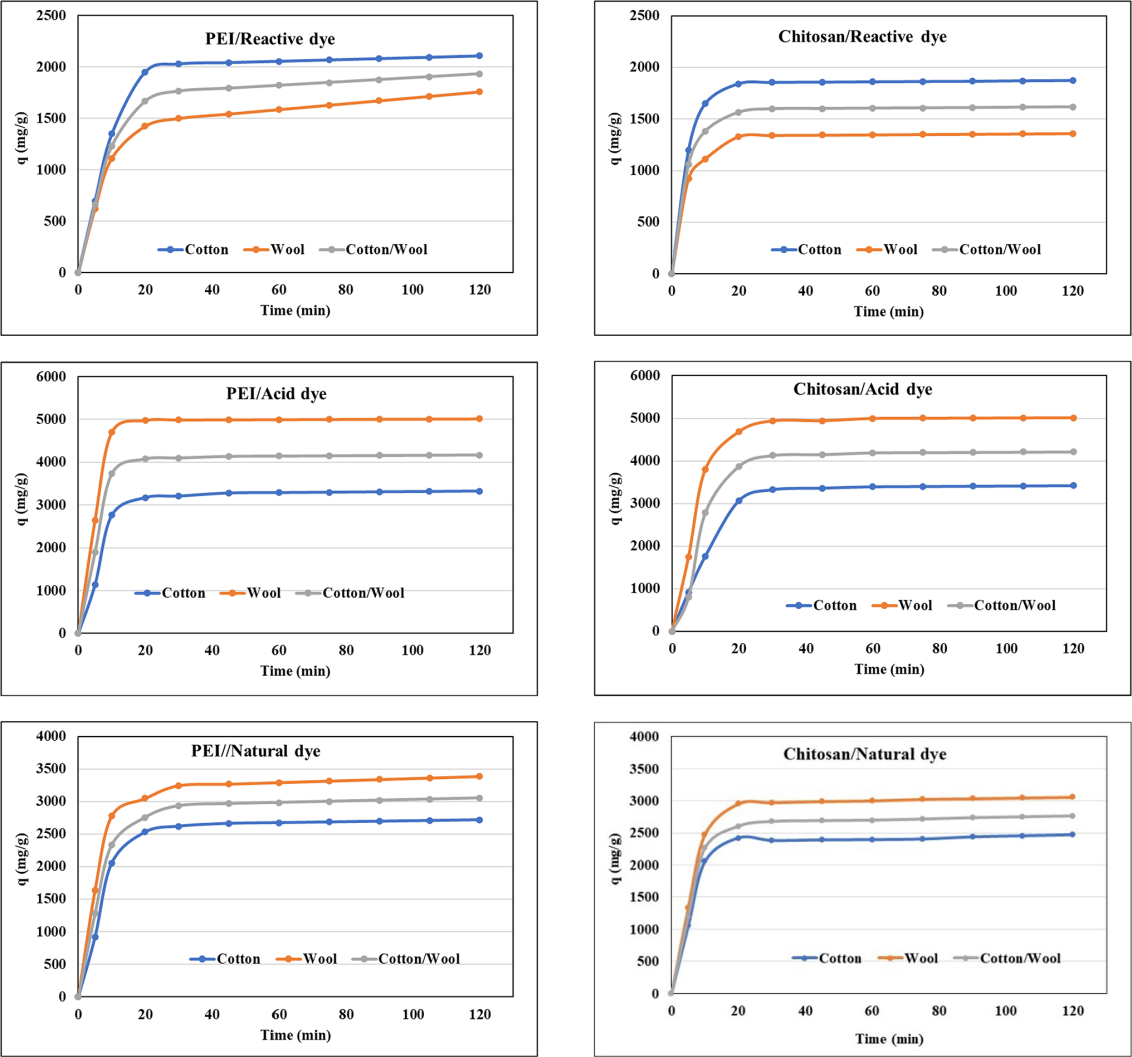
Effect of time on the adsorption of different dyes nature onto different treated fabrics with PEI and Chitosan at 25 °C.

Contact time is the amount of time it takes for the adsorption process to achieve equilibrium^70^. The effects of contact duration on dye adsorption on the treated fabrics using polycation compounds were examined. Figure 10 demonstrates that dye’s adsorption increased significantly from 1 to 20 min and that the equilibrium for all treated fabrics was achieved in 20–120 min. In general, all dyes are trending in the same behavior. The adsorption rate of dyes was initially greater due to the presence of a large number of active sites for adsorption onto the fabric surface. The adsorption rate then steadily dropped owing to a decrease in the number of adsorption active sites until equilibrium was attained.

### Adsorption isotherms

At constant temperature, the adsorption isotherm may describe the interaction and equilibrium connection between adsorbed solution and adsorbent^71^. The evaluation of isotherm data by fitting it to several isotherm models is a critical step in determining the best appropriate model to represent the adsorption process^72,73^. Adsorption data like Langmuir and Freundlich isotherms are commonly represented as adsorption models^74,75^. The Langmuir model explains the adsorption of adsorbate onto an adsorbent surface, which results in the formation of a monolayer on the adsorbent’s outer surface. It is assumed that adsorption sites are confined and homogeneous^76^. The equation that represents the Langmuir isotherm is as follows:^77^

$$\:\frac{{C}_{e}}{{q}_{e}}=\frac{1}{\left({q}_{max}\bullet\:{K}_{L}\right)}+\frac{{C}_{e}}{{q}_{\text{max}\:}}\:$$

where C_e_ denotes the dye concentration in an aqueous solution at equilibrium, q_e_ denotes the equilibrium concentration of dyes adsorbed by the treated fabrics, q_max_ denotes the fabric’s adsorption capacity for monolayer formation, and K_L_ denotes the Langmuir constant, which is related to the apparent energy of adsorption. On a plot of C_e_/q_e_ versus C_e_, a straight line with a slope of (1/q_max_) and an intercept of [1/(KL q_max_)] should be calculated. The basic properties of the Langmuir isotherms may be expressed by a separation factor (R_L_), which is described by the equation:^78^

$$\:{R}_{L}=\frac{1}{1\:+\:\left({K}_{L}\bullet\:{C}_{e}\right)}$$

where R_L_ values denote (a) irreversible [if R_L_= 0], (b) favorable [if (0 < R_L_ < 1)], (c) linear [if R_L_= 1], or (d) unfavorable [if R_L_ > 1] adsorption. This is the most often used model for isothermal adsorption, and it agrees well with many experimental data^79,80^.

**Fig. 11:**
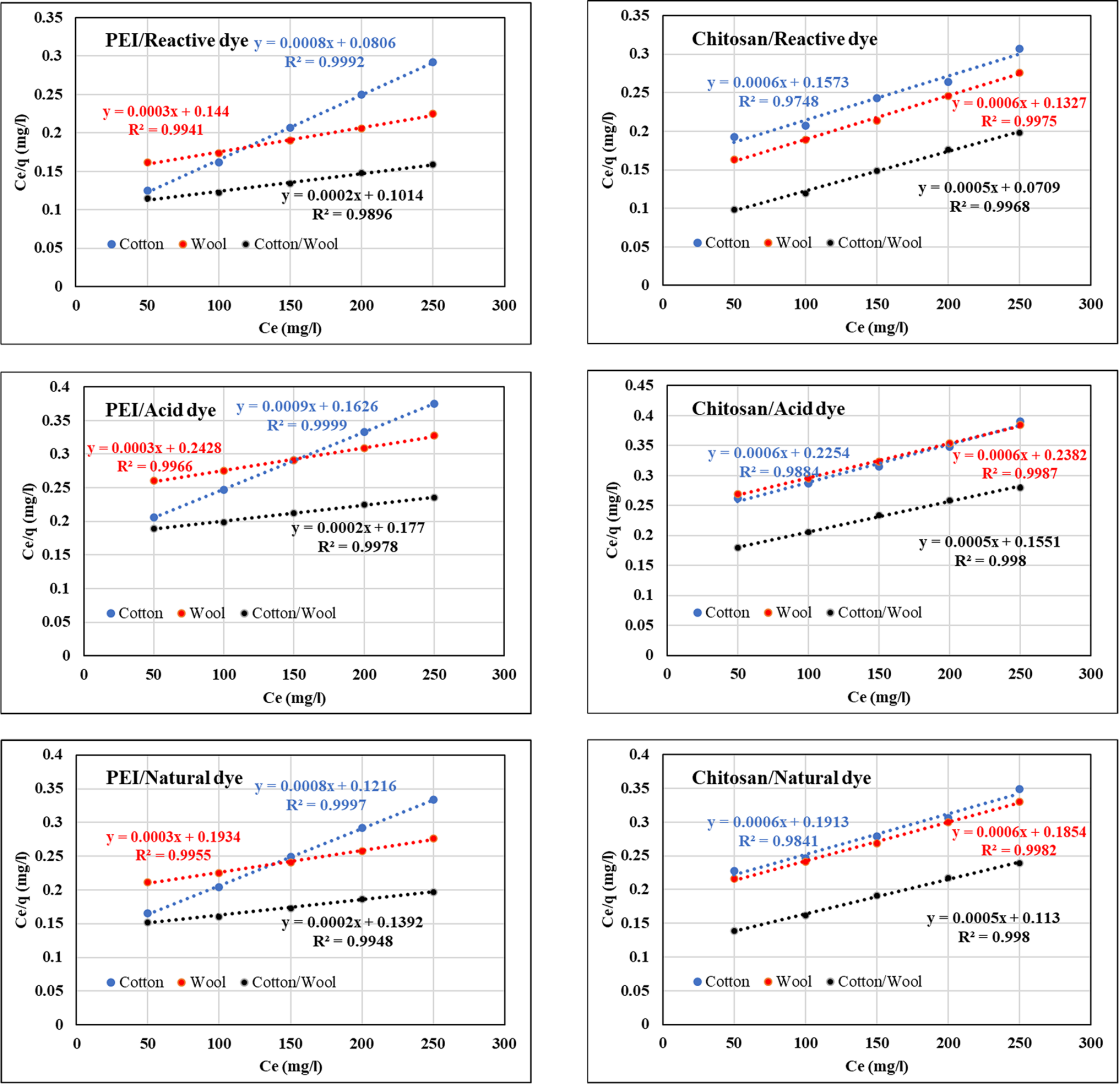
Langmuir adsorption of different dyes nature onto different treated fabrics with PEI and Chitosan at 25 °C.

The Langmuir adsorption isotherms of investigated dyes adsorbed onto treated fabrics (cotton, wool, and cotton/wool) with both polycation compounds (Chitosan and PEI) are plotted in Fig. 11, and the isothermal constants, q_max_, K_L_, R_L_, and the coefficient of correlation (R^2^) are listed in Table 8. The Langmuir constant (K_L_) gives information on the intensity of interactions between adsorbed and absorbed molecules. The greater the interaction, the higher the K_L_ value, and vice versa. As demonstrated in Table 8, the K_L_ value for all treated fabrics is very low, implying a poor interaction between these treated fabrics and dyes^81^. Calculating the R_L_ for all treated fabrics with both polycation compounds which ranged from 0 to 1, indicated that all investigated dyes adsorb well^82^. In addition, the R^2^ for reactive dyes onto all investigated fabrics with both polycations was closer to unity, indicating a good mathematical match. As the active sites of the polycations surface are homogeneously distributed, the adsorption of dyes onto the treated fabrics is consistent with the Langmuir isothermal model under the applied optimal circumstances^83^.

While the Freundlich model, which is based on the fact that multilayer adsorption happens on a heterogeneous surface and assumes that adsorption occurs at sites with varied adsorption energy^84^, implies that adsorption occurs at sites with varying adsorption energy. The Freundlich isotherm is denoted by the following equation:^85^

$$\:\text{ln}{q}_{e\:}=\:\text{ln}{K}_{F}\:+\:\frac{1}{n}\:\text{ln}{C}_{e}$$

The quantity of dyes adsorbed at equilibrium concentration is represented by K_F_ (also known as the Freundlich adsorption constant), whereas 1/n is the adsorption intensity or surface heterogeneity (Freundlich parameter). Where 1/n values denote (a) irreversible [if 1/*n* = 0], (b) favorable [if (0 < 1/*n* < 1)], or (c) unfavorable [if 1/*n* > 1] adsorption.

**Fig. 12:**
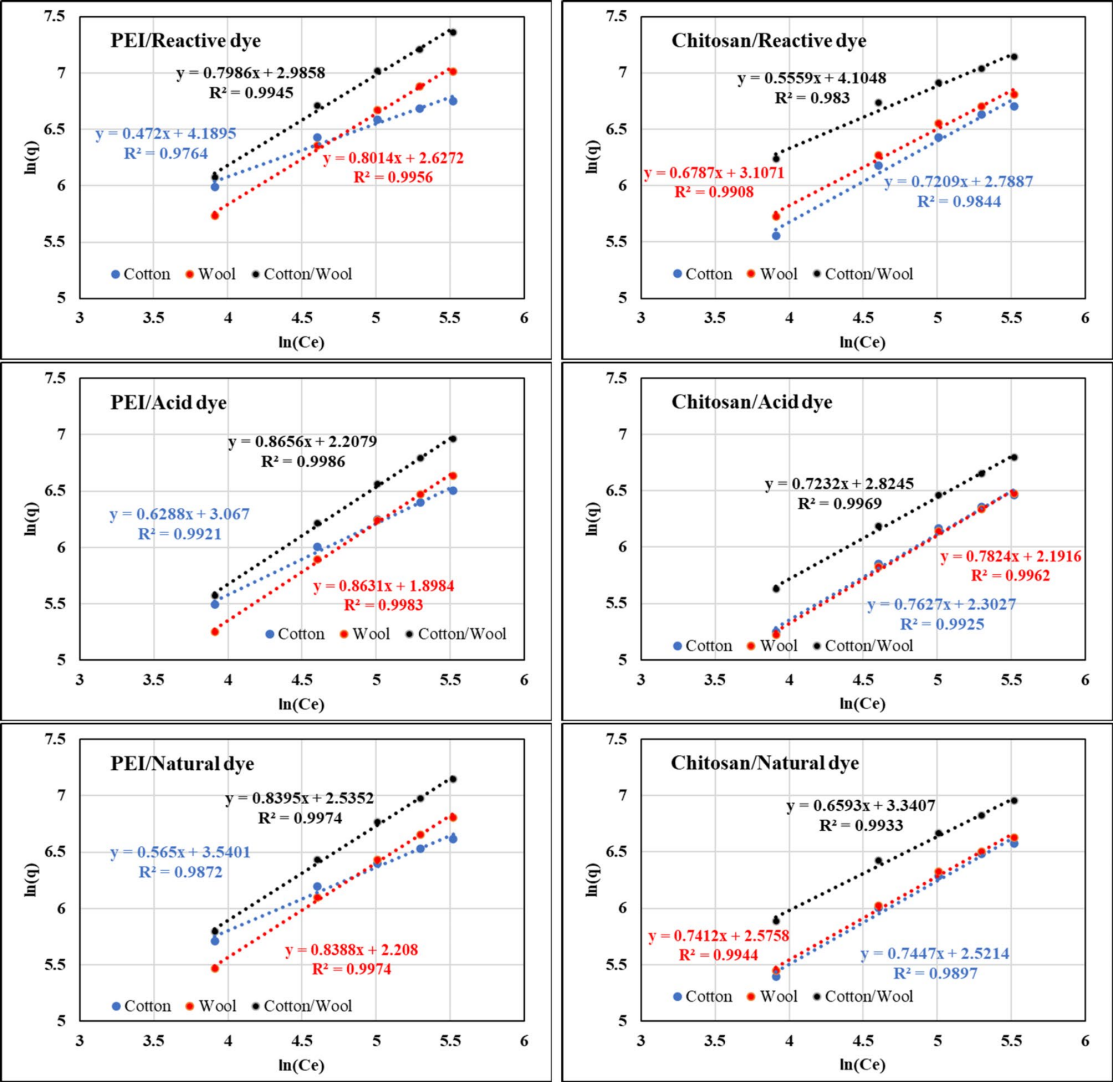
Freundlich adsorption of different dyes nature onto different treated fabrics with PEI and Chitosan at 25 °C.

When 1/n is smaller than 1, a chemical reaction is involved in the adsorption. Adsorption is a physical mechanism that otherwise occurred^86^. The K_F_ and 1/n values were calculated by plotting ln q_e_ vs. ln C_e_ from the intercept and slope, respectively, as shown in Fig. 12; Table 9. The 1/n values are less than one and higher than 0, indicating that the adsorption process is favorable. Furthermore, the 1/n values for dyes in all of these investigations were between 0.1 and 1, showing that chemisorption was preferred over the adsorption of investigated dyes^87^. If the R^2^ value approaches unity, the adsorption process is considered to agree with a certain isotherm model, such as the Langmuir or Freundlich isothermal model. As can be seen from the results in Table 8 and Table 9, dye adsorption by each treated fabric with both polycation compounds follows the Langmuir isotherm model rather than the Freundlich isotherm model.

### Kinetics of dyes adsorption

Three alternative kinetic models, including pseudo-first-order, pseudo-second-order, and intra-particle diffusion, were used to study dye adsorption by the treated fabrics with both polycation compounds in terms of adsorption kinetics, and experimental results were introduced using the following equations:^88^

$$\:\text{ln}\left({q}_{1}-{q}_{t}\right)=\text{ln}{q}_{1}-{K}_{1}t\:\:\:\:\text{p}\text{s}\text{e}\text{u}\text{d}\text{o}-\text{f}\text{i}\text{r}\text{s}\text{t}-\text{o}\text{r}\text{d}\text{e}\text{r}$$

where *q*_*t*_ is the amount of dye adsorbed onto treated fabrics at various times *t*, *q*_*1*_ is the amounts of dye adsorbed onto treated fabrics at equilibrium, and *k*_*1*_*is* the adsorption rate constants of the pseudo-first-order model.

$$\:\frac{\text{t}}{{\text{q}}_{\text{t}}}=\frac{1}{{\text{K}}_{2}{\text{q}}_{2}^{2}}+\left(\frac{1}{{\text{q}}_{2}}\right)\text{t}\:\:\text{p}\text{s}\text{e}\text{u}\text{d}\text{o}-\text{s}\text{e}\text{c}\text{o}\text{n}\text{d}-\text{o}\text{r}\text{d}\text{e}\text{r}$$

where *q*_*t*_ is the amount of dye adsorbed onto treated fabrics at various times *t*, *q*_*2*_ is the amounts of dye adsorbed onto treated fabrics at equilibrium, and *k*_*2*_*is* the adsorption rate constants of the pseudo-second-order model.

$\:{\text{q}}_{\text{t}}={\text{k}}_{\text{P}}{\text{t}}^{1/2}+\text{C}$ intra-particle diffusion

where *q*_*t*_ is the amount of dye adsorbed onto treated fabrics at various times *t*, and *k*_*p*_*is* the adsorption rate constants of the intra-particle diffusion model.

**Fig. 13:**
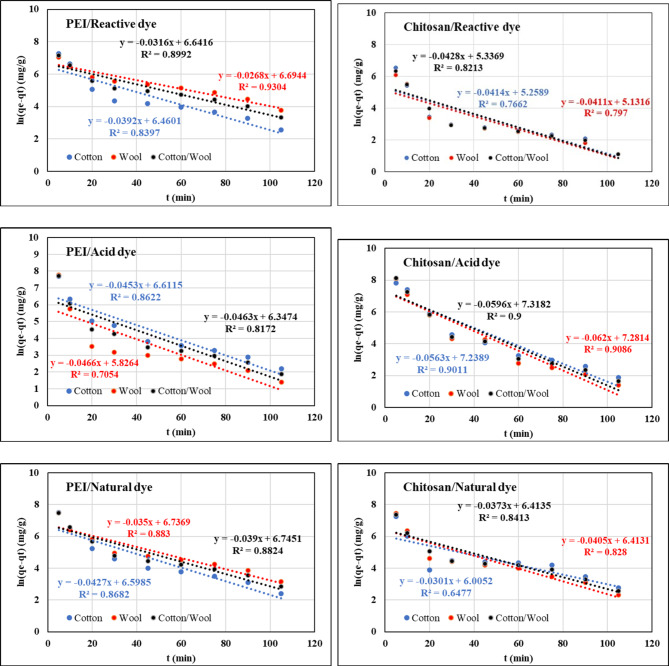
Kinetic pseudo-first-order isotherm of different dyes nature onto different treated fabrics with PEI and Chitosan at 25 °C.

**Fig. 14:**
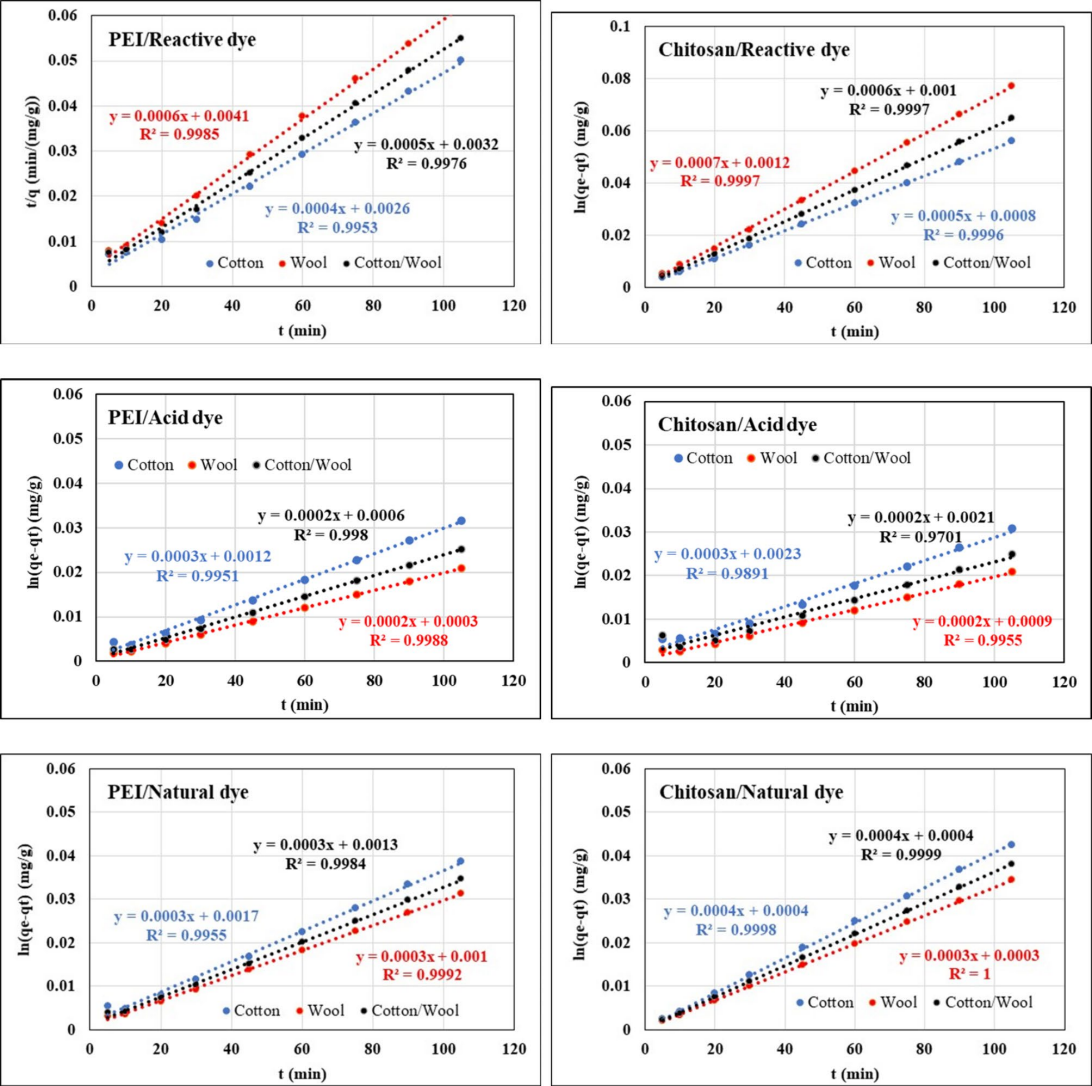
Kinetic pseudo-second-order isotherm of different dyes nature onto different treated fabrics with PEI and Chitosan at 25 °C.

**Fig. 15:**
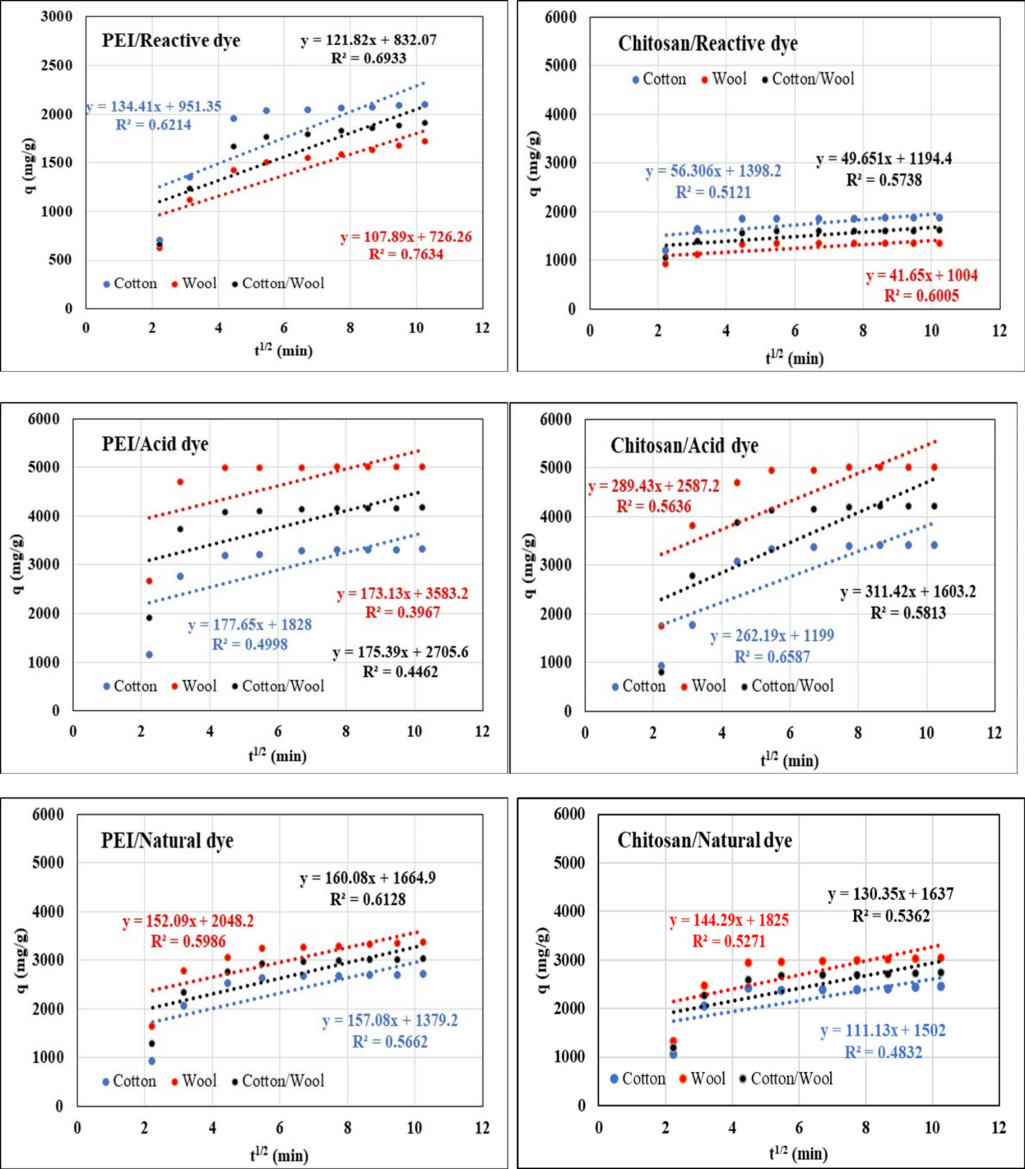
Kinetic Intra particle isotherm of different dyes nature onto different treated fabrics with PEI and Chitosan at 25 °C.

The results of the experimental data for the adsorption of each dye onto treated fabrics of these models are presented in Figs. 13, 14, and 15. Tables 10, 11, and 12 summarize the kinetic parameters of kinetic models. The experimental data’s fit to the kinetic models was assessed using a high correlation coefficient (R^2^) and close agreement between computed and experimental values (q_1_ for the pseudo-second-order model). For all of the treated fabrics for each investigated dyes, the evident and strong divergence between the measured absorption capacity and the estimated absorption capacity suggests compatibility with pseudo-second-order kinetics. In comparison to the pseudo-first-order kinetic model, the theoretical equilibrium adsorption capacities obtained from the pseudo-second-order model was very well associated with the experimental results and had better correlational coefficients (R^2^ > 0.999) values, indicating that the second-order model suited well^89,90^. Furthermore, physical adsorption is also the rate-determination phase, according to the pseudo-second-order kinetic model. In addition, if intraparticle diffusion is the primary rate control step, the graph should be linear and pass through the origin, according to its equation. All dyes plots did not pass through the origin, as illustrated in Fig. 15, indicating that no zero intercept was discovered. The C’s constant values were also non-zero.

**Table 8:** Langmuir isotherm parameters for adsorption of dyes by treated fabrics.

Polycation	Dye	Langmuir parameters	Treated Fabrics		
			Cotton	Wool	Cotton/Wool
PEI	Reactive	*q*_*max*_ (mg/g)	1250	3333.333333	5000
		*K*_*L*_	0.00006448	0.0000432	0.00002028
		*R*_*L*_	0.756200847	0.822368421	0.907935355
		*R* ^*2*^	0.9992	0.9941	0.9896
	Acid	*q*_*max*_ (mg/g)	1111.111111	3333.333333	5000
		*K*_*L*_	0.00014634	0.00007284	0.0000354
		*R*_*L*_	0.577467229	0.733030347	0.849617672
		*R* ^*2*^	0.9999	0.9966	0.9978
	Natural	*q*_*max*_ (mg/g)	1250	3333.333333	5000
		*K*_*L*_	0.00009728	0.00005802	0.00002784
		*R*_*L*_	0.672766416	0.775133711	0.877808989
		*R* ^*2*^	0.9997	0.9955	0.9948
Chitosan	Reactive	*q*_*max*_ (mg/g)	1666.666667	1666.666667	2000
		*K*_*L*_	0.00009438	0.00007962	0.00003545
		*R*_*L*_	0.679393981	0.715256419	0.849437248
		*R* ^*2*^	0.9748	0.9975	0.9968
	Acid	*q*_*max*_ (mg/g)	1666.666667	1666.666667	2000
		*K*_*L*_	0.00013524	0.00014292	0.00007755
		*R*_*L*_	0.596587519	0.583226408	0.720590885
		*R* ^*2*^	0.9884	0.9987	0.998
	Natural	*q*_*max*_ (mg/g)	1666.666667	1666.666667	2000
		*K*_*L*_	0.00011478	0.00011124	0.0000565
		*R*_*L*_	0.635364381	0.642590927	0.779727096
		*R* ^*2*^	0.9841	0.9982	0.998

**Table 9:** Freundlich isotherm parameters for adsorption of dyes by treated fabrics.

Polycation	Dye	Freundlich Parameters	Treated Fabrics		
			Cotton	Wool	Cotton/Wool
PEI	Reactive	*K*_*F*_	65.98978782	13.83497768	19.80233777
		*1/n*	0.4720	0.8014	0.7986
		*R* ^*2*^	0.9764	0.9956	0.9945
	Acid	*K*_*F*_	21.477374	6.67521	9.09659
		*1/n*	0.6288	0.8631	0.8656
		*R* ^*2*^	0.9921	0.9983	0.9986
	Natural	*K*_*F*_	34.470366	9.0975	12.619
		*1/n*	0.5650	0.8388	0.8395
		*R* ^*2*^	0.9872	0.9974	0.9974
Chitosan	Reactive	*K*_*F*_	16.2599	22.3561	60.63062
		*1/n*	0.7209	0.6787	0.5559
		*R* ^*2*^	0.9844	0.9908	0.983
	Acid	*K*_*F*_	10.0011	8.94952	16.85252
		*1/n*	0.7627	0.7824	0.7232
		*R* ^*2*^	0.9925	0.9962	0.9969
	Natural	*K*_*F*_	12.446	13.1418	28.23889
		*1/n*	0.7447	0.7412	0.6593
		*R* ^*2*^	0.9897	0.9944	0.9933

**Table 10:** Kinetic pseudo-first-order isotherm parameters for adsorption of dyes by treated fabrics.

Polycation	Dye	Pseudo-first-order parameters	Treated Fabrics		
			Cotton	Wool	Cotton/Wool
PEI	Reactive	*q*_*max*_ (Exp.) (mg/g)	2109	1759	1934
		*q*_*max*_ (Cal.) (mg/g)	639.1249659	807.8690673	766.3201249
		*K*_*1*_ (min^− 1^)	0.0392	0.0268	0.0316
		*R* ^*2*^	0.8397	0.9304	0.8992
	Acid	*q*_*max*_ (Exp.) (mg/g)	3327	5013	4170
		*q*_*max*_ (Cal.) (mg/g)	743.597579	339.1355907	571.0061613
		*K*_*1*_ (min^− 1^)	0.0453	0.0466	0.0463
		*R* ^*2*^	0.8622	0.7054	0.8172
	Natural	*q*_*max*_ (Exp.) (mg/g)	2718	3386	3052
		*q*_*max*_ (Cal.) (mg/g)	733.993373	842.94356	849.88411
		*K*_*1*_ (min^− 1^)	0.0427	0.035	0.035
		*R* ^*2*^	0.8682	0.883	0.8824
Chitosan	Reactive	*q*_*max*_ (Exp.) (mg/g)	1873	1359	1616
		*q*_*max*_ (Cal.) (mg/g)	192.26988	169.28776	207.8673
		*K*_*1*_ (min^− 1^)	0.0414	0.0411	0.0428
		*R* ^*2*^	0.7662	0.797	0.8213
	Acid	*q*_*max*_ (Exp.) (mg/g)	3420	5013	4216.5
		*q*_*max*_ (Cal.) (mg/g)	1392.5613	1453.0208	1507.488
		*K*_*1*_ (min^− 1^)	0.0563	0.062	0.0596
		*R* ^*2*^	0.9011	0.9086	0.9
	Natural	*q*_*max*_ (Exp.) (mg/g)	2468	3054	2761
		*q*_*max*_ (Cal.) (mg/g)	405.53209	609.78108	610.025
		*K*_*1*_ (min^− 1^)	0.0301	0.0405	0.0373
		*R* ^*2*^	0.6477	0.828	0.8413

**Table 11:** Kinetic pseudo-second-order isotherm parameters for adsorption of dyes by treated fabrics.

Polycation	Dye	Pseudo-second-order parameters	Treated Fabrics		
			Cotton	Wool	Cotton/Wool
PEI	Reactive	*q*_*max*_ (Exp.) (mg/g)	2109	1759	1934
		*q*_*max*_ (Cal.) (mg/g)	2500	1666.666667	2000
		*K*_*2*_ (g/mg.min)	6.15385E-05	8.78049E-05	0.000078125
		*R* ^*2*^	0.9953	0.9985	0.9976
	Acid	*q*_*max*_ (Exp.) (mg/g)	3327	5013	4170
		*q*_*max*_ (Cal.) (mg/g)	3333.333333	5000	5000
		*K*_*2*_ (g/mg.min)	0.000075	0.000133333	6.66667E-05
		*R* ^*2*^	0.9951	0.9988	0.998
	Natural	*q*_*max*_ (Exp.) (mg/g)	2718	3386	3052
		*q*_*max*_ (Cal.) (mg/g)	3333.333333	3333.333333	3333.333333
		*K*_*2*_ (g/mg.min)	5.29412E-05	0.00009	6.92308E-05
		*R* ^*2*^	0.9955	0.9992	0.9984
Chitosan	Reactive	*q*_*max*_ (Exp.) (mg/g)	1873	1359	1616
		*q*_*max*_ (Cal.) (mg/g)	2000	1428.571429	1666.666667
		*K*_*2*_ (g/mg.min)	0.0003125	0.000408333	0.00036
		*R* ^*2*^	0.9996	0.9997	0.9997
	Acid	*q*_*max*_ (Exp.) (mg/g)	3420	5013	4216.5
		*q*_*max*_ (Cal.) (mg/g)	3333.333333	5000	5000
		*K*_*2*_ (g/mg.min)	3.91304E-05	4.44444E-05	1.90476E-05
		*R* ^*2*^	0.9891	0.9955	0.9701
	Natural	*q*_*max*_ (Exp.) (mg/g)	2468	3054	2761
		*q*_*max*_ (Cal.) (mg/g)	2500	3333.333333	2500
		*K*_*2*_ (g/mg.min)	0.0004	0.0003	0.0004
		*R* ^*2*^	0.9998	1	0.9999

**Table 12:** Kinetic intra particle isotherm parameters for adsorption of dyes by treated fabrics.

Polycation	Dye	Intra particle parameters	Treated Fabrics		
			Cotton	Wool	Cotton/Wool
PEI	Reactive	K_P_	134.41	107.89	121.82
		C	951.35	726.26	832.07
		*R* ^*2*^	0.6214	0.7634	0.6933
	Acid	K_P_	177.65	173.13	175.39
		C	1828	3583.2	2705.6
		*R* ^*2*^	0.4998	0.3967	0.4462
	Natural	K_P_	157.08	152.09	160.08
		C	1379.2	2048.2	1664.9
		*R* ^*2*^	0.5662	0.5986	0.6128
Chitosan	Reactive	K_P_	56.306	41.65	49.651
		C	1398.2	1004	1194.4
		*R* ^*2*^	0.5121	0.6005	0.5738
	Acid	K_P_	262.19	289.43	311.42
		C	1199	2587.2	1603.2
		*R* ^*2*^	0.6587	0.5636	0.5813
	Natural	K_P_	111.13	144.29	130.35
		C	1502	1825	1637
		*R* ^*2*^	0.4832	0.5271	0.5362

##  Conclusion

Polycation modification of textiles materials (cotton, wool, and cotton/wool fabrics) was investigated. The color performance of textile fabrics dyed with the different dye nature (reactive, acid, and natural (tea extract) dyes) has improved as a result of both treatments with both polycation compounds, as the fabric dyeability, color strength K/S and colorfastness properties were enhanced. Furthermore, the treated fabrics gained a new functional property (antimicrobial protection), besides saving the required amount of dyestuff and chemicals which consequent save costs and causes less environmental pollution, and also time and temperature that are required for the dyeing process, as the optmium conditions for this study is 1% for the polycation concentration, 20 min for the dyeing time and 75 °C for the dyeing temperature.

A decrease in dye concentration (from 2 to 0.5 g/L), dyeing temperature (from 90 to 75 °C), and dyeing time (from 90 to 20 min) has also been proved compared to traditional dyeing conditions.

Finishing of cotton/wool with PEI and Ch helped to obtain a high color homogeneity of the color resulting from dyeing in contrast to untreated samples, as well as overcoming the problem of the appropriate pH difference for each substrate separately, as the dyeing process was carried out in one step without the need to add acid or alkali but the dyeing bath included only the treated substrate and the dye solution. The FTIR spectra demonstrated the efficient adherence of polycations to the surface of cotton and wool fabrics. This simple and environmentally friendly method might be a viable alternative to the traditional cotton natural dyeing procedure.

## Data Availability

The datasets used and/or analysed during the current study available from the corresponding author on reasonable request.
